# Digital Cognitive Phenotyping for Differential Diagnosis and Monitoring in Neurological Conditions

**DOI:** 10.1002/acn3.70451

**Published:** 2026-07-11

**Authors:** Martina Del Giovane, Valentina Giunchiglia, Michael C. B. David, Magdalena A. Kolanko, William R. Trender, Peter J. Hellyer, Harmeena Kaur, David J. Sharp, Christopher Carswell, Paresh A. Malhotra, Adam Hampshire

**Affiliations:** ^1^ Department of Brain Sciences Imperial College London London UK; ^2^ UK Dementia Research Institute Care Research and Technology Centre, Sir Michael Uren Hub Imperial College London London UK; ^3^ Department of Neuroimaging, Institute of Psychiatry, Psychology and Neuroscience King's College London London UK; ^4^ Department of Biomedical Informatics Harvard Medical School Boston Massachusetts USA; ^5^ Department of Neurology Imperial College Healthcare NHS Trust, Charing Cross Hospital London UK

**Keywords:** Alzheimer's disease, dementia, normal pressure hydrocephalus, online cognitive testing, traumatic brain injury

## Abstract

**Objective:**

To assess the utility, accessibility, and equivalence to supervised scales of online cognitive assessment in older individuals with cognitive impairment.

**Methods:**

Patients with Alzheimer's disease (AD, *n* = 31), idiopathic normal pressure hydrocephalus (iNPH, *n* = 26), and traumatic brain injury (TBI, *n* = 23) completed online cognitive tasks (Cognitron). We evaluated cognition relative to a large normative dataset (*N* ≈ 400,000), adjusting for device and demographics which can affect performance. Principal Component Analysis (PCA) was used to derive domain‐specific and total composite scores. We compared clinical groups and correlated performance with standard assessments.

**Results:**

Uptake was ~70%. PCA identified components across memory, processing speed, language, and executive functions. AD showed memory and language impairments compared with the norms and other groups. iNPH had greater executive and processing speed deficits, consistent with a subcortical impairment profile. TBI showed milder deficits in memory, working memory, and language. Cognitron total composite was associated with standard supervised tests (ADAS‐Cog: *β* = −0.76, *p* < 0.001 and ACE‐III: *β* = 0.69, *p* < 0.001). In iNPH, Cognitron composite predicted walking speed (estimate = 1.10, *p* < 0.001), a core clinical feature of the disease which is difficult to evaluate remotely. We selected five tasks with high completion rates, discriminability between conditions, and broad cognitive coverage. The derived short composite showed very high accuracy in separating AD (AUC = 0.94) and iNPH (AUC = 0.90) from age‐matched norms; performance was weaker for TBI (AUC = 0.66).

**Interpretation:**

Online assessment in older clinical populations is feasible and sensitive to subtle disease‐specific cognitive deficits. A demographically adjusted, 15‐min battery offers a scalable adjunct to standard testing, with potential to reduce burden on patients and healthcare systems.

## 1 Introduction

Cognitive testing is central to assessing those with or at risk of developing dementia. Paper‐based tests are gold‐standard, but are limited by practical constraints, including the need for trained administrators, in‐person attendance, and time and financial costs. Cognitive examination is often conducted long after the onset of symptoms [1]. When it occurs, the use of a range of different tools limits interpretability [2, 3].

Online cognitive assessments are a scalable and accessible alternative, offering the possibility of harmonising measurement across stages and enabling continuous, flexible cognitive profiling. The capture of detailed measures enhances sensitivity to subtle cognitive differences. This makes them useful not only for measuring early‐stage deficits [4], but also for assessing overlapping clinical conditions [5] and tracking disease progression and treatment response [6]. Recent initiatives have sought to define best practices and confirm the clinical utility of in‐person digital assessments for people with cognitive concerns or at risk of developing dementia [7]. However, the development of online assessments for older patients with existing neurological diagnosis presents additional challenges. Some have had less exposure to technology whilst the nature of cognitive impairment can compromise their ability to understand and complete some tasks [8]. There is also the need for brief assessments that minimise patient burden yet have sufficient detail to differentiate between disorders. Finally, it is important to interpret performance in relation to gold‐standard supervised assessments. These considerations underscore the need for studies that tailor and validate computerised assessments in this context.

We aimed to develop a brief, automated cognitive battery that is (a) applicable across different neurological conditions and levels of disease severity; (b) usable across different clinical scenarios including differential diagnosis and monitoring of cognitive abilities; and (c) deliverable both in supervised and unsupervised settings. This is done via assessing the validity and sensitivity of a set of online tasks in patients with Alzheimer's disease (AD), traumatic brain injury (TBI) and idiopathic normal pressure hydrocephalus (iNPH). Since these conditions can produce overlapping cognitive deficits and require cognitive tracking, they are ideal for validating a scalable, transdiagnostic and remotely deployable battery. AD typically begins with progressive episodic and semantic memory loss, though atypical variants may affect language, visuospatial, or executive functions depending on cortical atrophy patterns [9]. Disease modifying therapies are currently being developed and have shown some beneficial effects at early disease stages [10]. Longitudinal cognitive tracking helps identify at‐risk individuals for early intervention, monitor progression and treatment response. In contrast, iNPH is caused by ventricular expansion and cerebrospinal fluid (CSF) accumulation [11], producing a frontal‐subcortical cognitive profile with slowed processing, executive dysfunction [12, 13], and memory deficits linked to executive rather than temporal lobe dysfunction [13, 14]. Its triad of gait disturbance, incontinence, and cognitive decline may improve with shunting surgery [15], but misdiagnosis with AD risks delaying or misdirecting treatment [16]. Ongoing monitoring is important, as cognitive gains can be delayed or relapse after surgery [17, 18]. TBI also produces persistent cognitive deficits and raises dementia risk [19, 20]. While rehabilitation can enable partial recovery [21], frontal‐network disruption often leads to executive, attentional, and memory problems [22], including post‐traumatic amnesia acutely and enduring episodic/prospective recall issues [23, 24]. As recovery can be nonlinear and heterogeneous, serial monitoring remains essential [25].

We deployed a battery of online cognitive tasks in individuals with AD, iNPH and moderate–severe TBI. Performance was adjusted for demographic factors using advanced modelling on a large‐scale normative dataset (*N* = 400,000). This also allowed correction for the device type, ensuring high device‐compatibility. We hypothesised that remote computerised testing would be feasible and clinically informative, reflected in high completion rates, alignment with standard assessments, and distinct impairment profiles across conditions.

## 2 Materials and Methods

### 2.1 Recruitment and Participants

Participants were recruited as part of three studies: (A) Minder, an ongoing longitudinal community‐based cohort study on individuals with a diagnosis of any form of dementia or mild cognitive impairment (MCI), led by the UK Dementia Research Institute Care Research and Technology Centre [26], (B) ‘Imperial Neurotrauma Centre Study of Clinical Outcomes after Traumatic Brain Injury’ [27], and (C) ‘Physiological Correlates of Noradrenergic Add‐on Therapy’ (PCNorAD), a study examining noradrenergic dysfunction in AD patients who have previously participated in the ‘NorAD’ clinical trial [28]. Recruitment sites were specialised memory clinics, general practitioner surgeries and a TBI clinic at St. Mary's Hospital, London. Participants were enrolled consecutively. TBI and iNPH patients were referred for enrolment following their appointment at specialist neurology clinics if they met the diagnostic criteria. AD patients were recruited through two routes: the same consecutive clinic‐based referral process, and separately, through attendance at Imperial College London as part of the Minder and PCNorAD studies, where eligible individuals were approached for consent to participate in the present study.

AD patients had an existing clinical diagnosis which was confirmed at a multidisciplinary review meeting including neurologists, psychiatrists, and neuroradiologists, and through assessment of clinical symptoms and biomarkers collected either as part of routine clinical care or within the wider Minder and PCNorAD studies. Regarding these biomarkers, we referred to those grouped by the National Institute on Ageing and the Alzheimer's Association into: (a) those directly reflecting AD pathology, such as positive amyloid or tau PET imaging and blood or CSF markers of tauopathy; and (b) those reflecting neurodegeneration that occurs in AD, such as brain atrophy quantified by MRI, and reduced glucose metabolism or perfusion [29]. Amyloid positivity was confirmed by amyloid PET scan in 12 AD participants and by CSF assessment in one subject. FDG‐PET findings were consistent with an AD pattern in two participants. All participants demonstrated evidence of neurodegeneration consistent with AD on MRI.

We categorised patients with radiological features consistent with iNPH, normal CSF opening pressure, gait impairment plus cognitive deficits or urinary incontinence, and a positive response to provocative testing (CSF tap test, lumbar infusion, or external lumbar drainage) as “probable iNPH” (*N* = 17) and those without a positive response to provocative testing as “possible iNPH” (*N* = 9) [11]. Three iNPH patients showed positive amyloid markers, two determined by CSF analysis and one determined by PET scan.

Moderate–severe TBI was defined according to the Mayo classification system, requiring the presence of one or more of the following: loss of consciousness of 30 min or more, post‐traumatic amnesia of 24 h or more, Glasgow Coma Scale score below 13, or neuroimaging evidence of intracranial injury such as intracerebral haematoma, subdural haematoma, cerebral contusion, haemorrhagic contusion, subarachnoid haemorrhage, brainstem injury, or penetrating dural injury [30]. All TBI participants were aged 50 or over to ensure age‐matching with the dementia groups.

**FIGURE 1:**
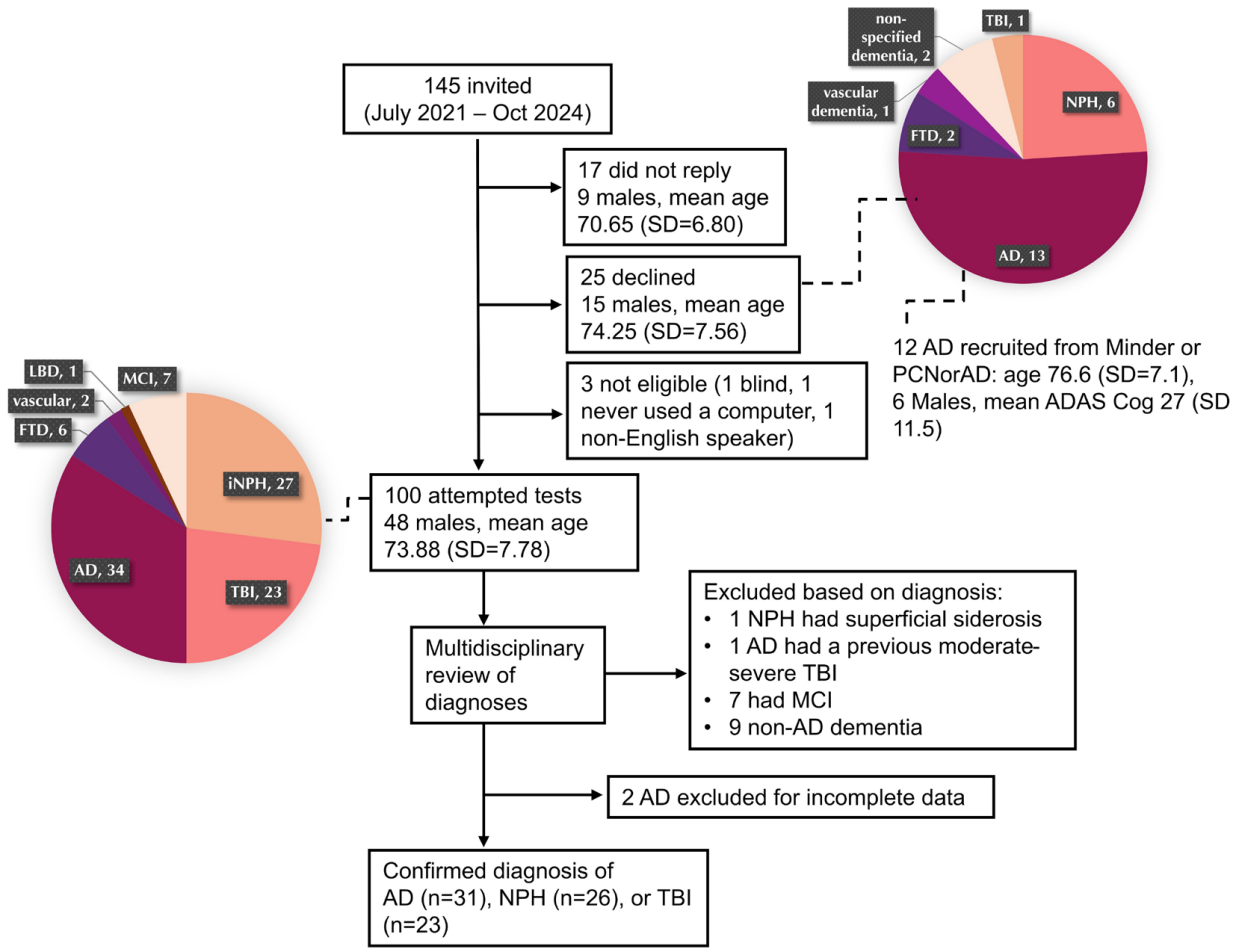
Recruitment flowchart. AD = Alzheimer's disease; FTD = frontotemporal dementia; iNPH = idiopathic normal pressure hydrocephalus; LBD = Lewy Body disease; MCI = mild cognitive impairment; TBI = traumatic brain injury.

A total of 145 individuals were invited to complete two sessions of online computerised cognitive assessments (Figure 1). Of these, 17 did not respond, 25 declined, and three were deemed ineligible. 100 remaining individuals attempted the tests. As the present analysis focuses on individuals with a confirmed diagnosis of either AD, TBI, or iNPH, seven individuals with MCI, six with frontotemporal dementia, two with vascular dementia, and one with Lewy body disease were excluded. After multidisciplinary review of clinical history, we excluded one iNPH participant due to the additional finding of superficial siderosis and one AD participant due to a previous moderate–severe TBI. Two additional individuals with AD withdrew after attempting the tasks.

### 2.2 Ethics Statement

The Minder study was approved by the Health Research Authority's London‐Surrey Borders Research Ethics Committee (19/LO/0102). The PCNorAD study was approved by the Health Research Authority's London‐Central Research Ethics Committee (18/LO/0249). The Imperial Neurotrauma Centre study was approved by the Camberwell St Giles Research Ethics Committee (17/l0/2066). The GBIT data collection received ethical approval by the Imperial College Research Ethics Committee (17IC4009). These studies were conducted in accordance with the Helsinki declaration. All participants gave written or electronic consent.

### 2.3 Cognitive Assessments

**FIGURE 2:**
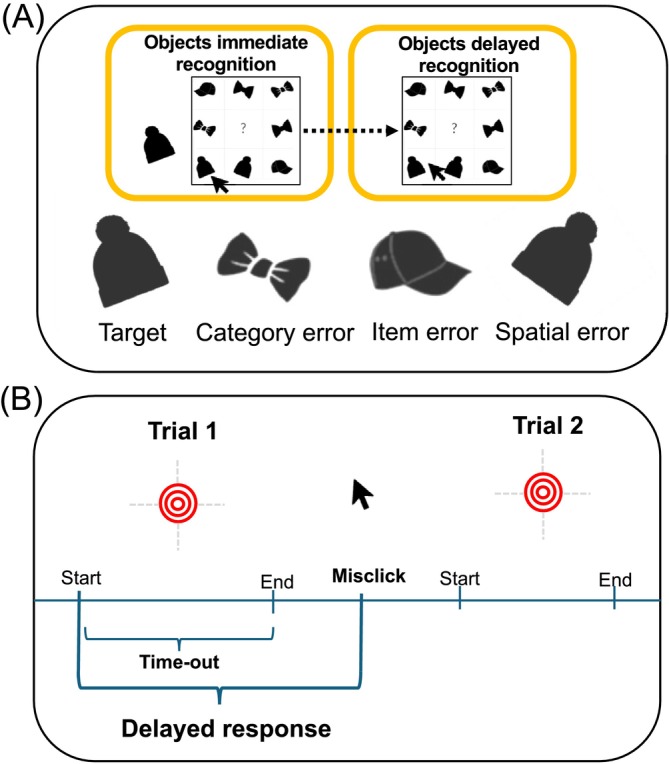
Cognitron tasks' details. (A) In the Objects Memory Recognition task, participants viewed 20 target objects, then identified them within 20 arrays of eight items each: One mirrored target, two same‐category items, and four unrelated distractors. Errors were classified as category error (unrelated distractor), item error (wrong same‐category item), or spatial error (correct item, wrong orientation). (B) Two signs of non‐compliance are recorded for Simple and Choice Reaction Time (RT): Time‐outs (no clicks during the target presentation) and mis‐clicks (clicks elsewhere, indicating disengagement). If a time‐out was immediately followed by a mis‐click, the RT was calculated as the time at which the ‘mis‐click’ occurred minus the start time of the trial where the ‘time‐out’ occurred.

Participants were asked to complete two online sessions of Cognitron tasks (https://www.cognitron.co.uk) [31, 32, 33]. Each session was accessed through a link sent via email, and all tasks were preceded by written and animated instructions. Each session covered multiple cognitive domains across Levels 1–3. Level 1 used simple rules; Level 2 required holding a rule in working memory and applying it to a task; Level 3 involved more complex reasoning and planning. Tasks were presented in increasing complexity to allow future auto‐skipping of difficult items. Sessions opened with an immediate memory recognition task and ended with a delayed one. Task descriptions and scoring are in Table S1. Most tasks generated a primary accuracy score and secondary RT score; for Simple Reaction Time (SRT), Choice Reaction Time (CRT), Trail Making, and Motor Control, RT was primary. Objects recognition tasks included additional accuracy measures (Figure 2A). SRT and CRT scores were mean RT across 60 trials. Non‐compliance was recorded as time‐outs (no clicks during target presentation) or mis‐clicks (clicks elsewhere), and trials containing at least one type were excluded. If a time‐out was followed by a mis‐click, RT was calculated from the start of the time‐out trial to the mis‐click to reflect a delayed response (Figure 2B).

To minimise fatigue, participants were encouraged to complete testing sessions on separate days, the intention being for the analyses reported here to guide selection of tasks that can be deployed in a single brief session. Participants were then asked to sit in a quiet room at home, review the on‐screen instructions independently, and notify their carer or a research assistant if they needed to pause or interrupt the assessment. Nine participants without a suitable device were provided with an Apple tablet for testing. Additionally, all participants completed the Alzheimer's Disease Assessment Scale‐Cognitive Subscale (ADAS‐Cog) in person (median time since completion of first Cognitron session = 0.72 months, SD = 0.9 months) [34]. For 27 ad and 25 iNPH patients, the Addenbrooke's Cognitive Examination‐III (ACE‐III) was retrieved from clinical records or prior studies (Minder and PCNorAD) [35]. The median time interval between completion of first session of Cognitron tasks and the ACE‐III was 2.25 months (SD = 4.47).

### 2.4 Statistical Analysis

Principal Component Analysis (PCA) with varimax rotation was applied to the Cognitron task summary scores. Composite scores were computed via Bartlett's regression. Component number was defined by the Kaiser criterion (eigenvalues > 1), along with the first unrotated component across all tasks to generate a total composite score (Global Cognition) and a short composite score from a subset of tasks. Due to the small patient sample, data were combined with an age‐matched subset of the Great British Intelligence Test (GBIT) normative dataset [36]. Missing task data were imputed using five‐iteration Markov Chain Monte Carlo (MCMC).

Individual accuracy, RT‐based, and composite scores were interpreted against the GBIT normative data [36]. Patients' scores were converted into Deviation from Expected (DfE) scores. Specifically, general linear models were trained on the normative dataset to predict the expected score of a cognitively healthy person with the same demographics (age, age^2^, gender, handedness, education, ethnicity, residency, first language, and device) as the patient. The DfE score describes the difference between the patient's actual raw score and the expected score, divided by the control population's SD.

As a DfE of 0 indicates the performance expected for an individual with the same demographics as the patient but no clinical diagnosis, cognitive impairment within each group was assessed using one‐sample *t*‐tests against 0, or Wilcoxon signed‐rank tests if the data were not normally distributed, with false discovery rate (FDR) correction applied. Between‐group differences were evaluated using one‐way ANOVA followed by pairwise *t*‐tests, or Kruskal–Wallis tests with post hoc Dunn's tests if residuals were non‐normal, with FDR correction for post hoc comparisons. Because cognitive scores were very low across groups, a simulated “pure guessing” participant was used to define task‐specific chance thresholds, and a sensitivity analysis excluded participants scoring below these thresholds (Supporting Information A).

We recommend a brief battery suitable across neurological conditions while minimising burden. Selection criteria were: (a) high completion rates, (b) strong discrimination between diagnostic groups or high loadings on distinguishing Cognitron components, and (c) coverage of multiple cognitive domains. Using PCA as described above, we derived a Cognitron short composite for these tasks and converted it to DfE. We then constructed receiver operating characteristic (ROC) curves for each clinical group and extracted the area under the curve (AUC) as a measure of discriminative performance between patients and the age‐matched subset of the GBIT normative dataset.

Finally, general linear models tested whether Cognitron total and short composites predicted ADAS‐Cog total score and ACE‐III total and sub‐scores. Robust regression was used if residuals were non‐normally distributed. Models also assessed prediction of iNPH gait time and velocity measured via the 10‐Meter Walking Test [37], which were retrospectively extracted from available clinical records. One wheelchair‐bound patient was assigned the maximum time and minimum velocity.

## 3 Results

### 3.1 Participants Demographics

The final study sample included 23 individuals with TBI (15 males, mean age = 69.87, SD = 9.45), 31 with AD (20 males, mean age = 74.26, SD = 8.71), and 26 with iNPH (18 males, mean age = 72.04, SD = 0.64). Time since TBI was 48.78 months (median = 35.02, IQR: 33.53). The mean ADAS‐Cog score was 27.90 (SD = 12.38) in AD, 22.22 (SD = 10.90) in iNPH and 14.74 (SD = 6.72) in TBI, with TBI performing significantly better than both AD and iNPH (*χ* ^2^(2) = 17.45, *p* < 0.001). Full demographics are summarised in Table S2.

### 3.2 Compliance and Uptake

The overall uptake across individuals with different types of dementia was 70%. There were no significant differences in age (*t* = 0.36, *p* = 0.72) or gender (*χ* ^2^(3) = 2.56, *p* = 0.46) between the 100 individuals who participated and those who did not, whether due to non‐response or declining the study invitation.

**FIGURE 3:**
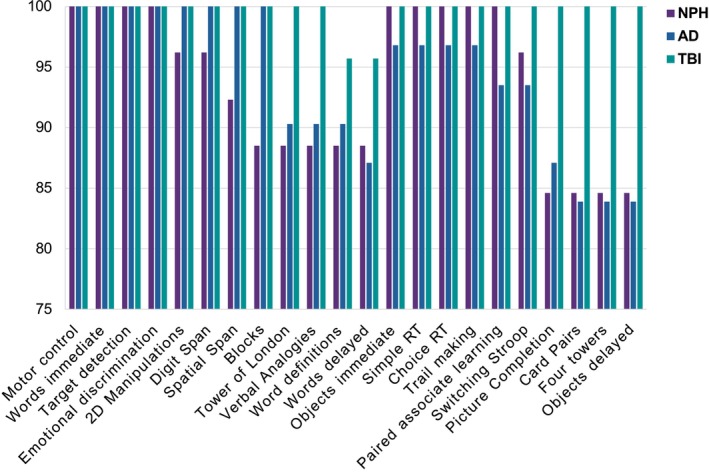
Completion rate in percentage for each cognitive task. The tasks are presented in sequential order, and the completion rate is calculated after exclusion of subjects showing signs of non‐compliance and excessively fast or slow responses. AD = Alzheimer's Disease; iNPH = idiopathic normal pressure hydrocephalus; TBI = traumatic brain injury; RT = reaction time.

Mean completion time was 32.84 min (SD = 10.59) for session one and 39.45 min (SD = 11.92) for session two, with no differences between AD, iNPH and TBI. Participants scoring 0 with no recorded RT were excluded from that task, indicating non‐attempt. Exclusions were: one AD participant (Target Detection), one AD and two iNPH participants (2D Manipulations), and one AD participant (PAL). Excessively long RTs were flagged using an IQR‐based rule and removed (Table S3). Task discontinuation increased at the third difficulty level. In battery one, completion rates were 89% (*n* = 23) for iNPH, 87% (*n* = 27) for AD, and 96% (*n* = 22) for TBI. In battery two, rates declined to 85% (*n* = 22) for iNPH and 84% for AD (*n* = 26), but remained 100% (*n* = 23) in TBI (Figure 3). Many participants showed unusually low DfE scores on Trail Making A; analyses therefore focused on Trail Making B. Possible explanations include familiarisation with the task during Trail Making A, caregiver support after observing difficulties on Trail Making A, and relatively slower performance on Trail Making B within the normative dataset, increasing the mean RT considered to be normal.

### 3.3 Cognitive Components of the Battery

PCA identified six interpretable components with eigenvalue > 1, which explained 54.60% of the variance (Figure S1). We defined these as *Objects Memory* (Objects Immediate and Delayed Recognition); *Words Memory* (Words Immediate and Delayed Recognition); *Processing Speed* (SRT, CRT, Motor Control, Trail Making, Target Detection, 2D Manipulations); *Language* (Verbal Analogies, Word Definitions, Digit Span); and two components representing higher order cognitive functions—one including Card Pairs, Spatial Span, Picture Completion, Paired Associate Learning, and Stroop (*Executive Functions I*); and one including Blocks, Emotion Discrimination, Four Towers, and Tower of London (*Executive Functions II*).

### 3.4 Correlation With Standard Clinical Assessments

**FIGURE 4:**
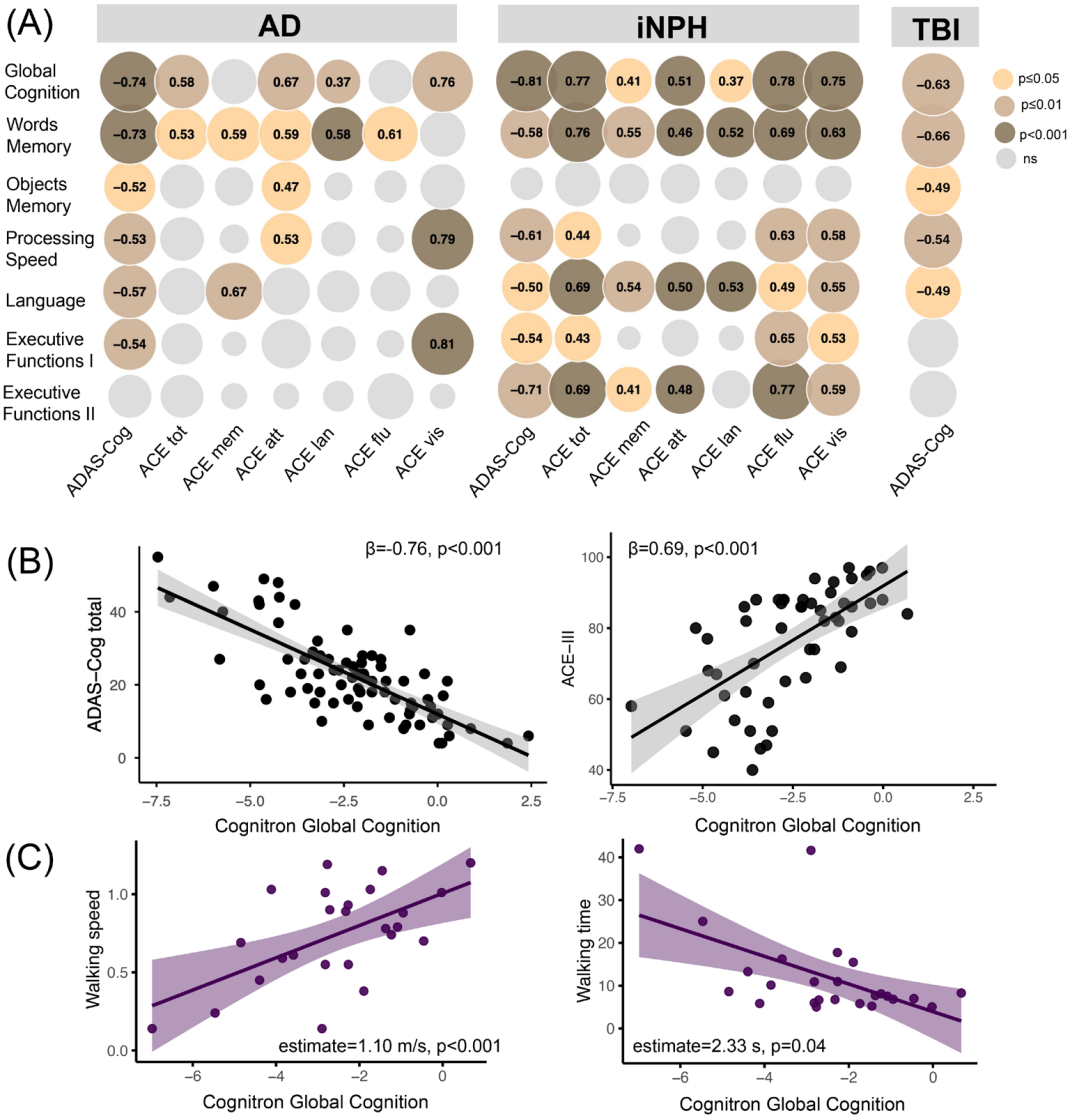
Associations of the Cognitron composites with standard cognitive measures using linear regression. (A) Bubble plots showing associations of the Cognitron composites with the ADAS‐Cog and the ACE‐III total scores and sub‐scores. The values and size of the bubbles correspond to the standardised *β*, while the colour reflects the significance level (FDR corrected within each Cognitron composite). ACE‐III scores were not available for the TBI group. (B) Scatter plot showing correlation of the Cognitron total composite (Global Cognition) with the ADAS‐Cog and ACE‐III total scores across all three groups of patients. (C) Scatter plots showing correlation of the Cognitron total composite with walking speed and time in the iNPH group. ACE‐III = Addenbrooke's Cognitive Examination‐III; ACE att = ACE attention sub‐score; ACE flu = ACE fluency sub‐score; ACE lan = ACE language sub‐score; ACE mem = ACE memory sub‐score; ACE vis = ACE visuospatial sub‐score; ADAS‐Cog = Alzheimer's Disease Assessment Scale‐Cognitive Subscale; AD = Alzheimer's disease; iNPH = idiopathic normal pressure hydrocephalus; TBI = traumatic brain injury.

As illustrated in Figure 4, the *Global Cognition* composite robustly predicted the ADAS‐Cog score in AD (*β* = −0.74, *p* < 0.001), iNPH (*β* = −0.81, p < 0.001), and TBI (*β* = −0.63, *p* = 0.001). Similarly, the ACE‐III scores increased with higher *Global Cognition* Cognitron composite in AD (*β* = 0.58, *p* = 0.01) and iNPH (*β* = 0.77, *p* < 0.001). In domain‐specific analyses, the Cognitron *Language* composite correlated with the ACE‐III Language sub‐score in iNPH (*β* = 0.53, *p* < 0.001) and with the ACE‐III memory sub‐score in AD (*β* = 0.67, *p* = 0.02). *Words Memory* was associated with the ACE‐III Memory sub‐score in AD (*β* = 0.59, *p* = 0.02) and iNPH (*β* = 0.55, *p* = 0.01). The Cognitron *Executive Functions* showed strong correlations with ACE‐III visuospatial in AD (*Executive Functions I*: *β* = 0.81, *p* < 0.001) and with ACE‐III fluency (*Executive Functions I* : *β* = 0.65, *p* = 0.003; *Executive Functions II*: *β* = 0.77, *p* < 0.001) and visuospatial (*Executive Functions I* : *β* = 0.53, *p* = 0.02; *Executive Functions II*: *β* = 0.59, *p* = 0.002) in iNPH. Full results are illustrated in Figure 4. Cognitron performance was also linked to functional outcomes. In iNPH, each one‐unit increase in *Global Cognition* was associated with a 2.33 s reduction in the time taken to walk 10 m (*p* = 0.04) and a 1.10 m/s increase in walking speed (*p* < 0.001) (Figure 4).

### 3.5 Cognitive Abnormalities on the Cognitron Composite Scores

**FIGURE 5:**
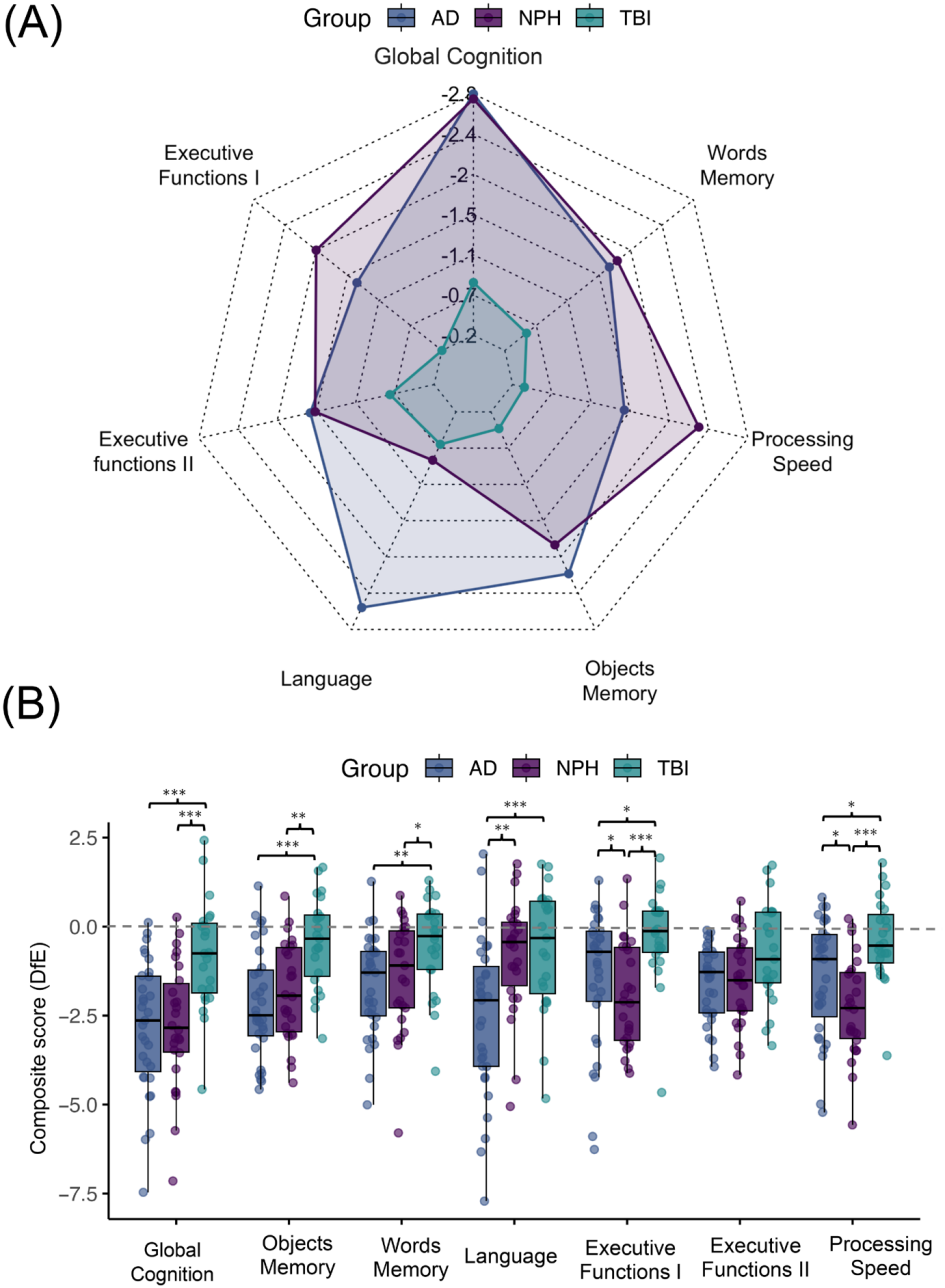
Performance on the Cognitron composite scores converted to Deviation from Expected (DfE) format. (A) Spider plot showing the cognitive profile of each clinical group. (B) Boxplot showing between‐group differences in performance. The dotted line represents the expected performance for an individual with the same demographic characteristics as the patient but no clinical diagnosis. *p ≤ 0.05, **p ≤ 0.01, ***p ≤ 0.001; AD = Alzheimer's disease; iNPH = idiopathic normal pressure hydrocephalus; TBI = traumatic brain injury.

*t*‐Tests against 0 indicated that AD and iNPH were significantly impaired across all cognitive domains relative to the normative dataset (Figure 5). Analyses of between‐group differences revealed that both AD and iNPH patients were more impaired than TBI on *Global Cognition* (*F*(2,77) = 9.74, *p* < 0.001), *Words Memory* (*χ* ^2^(2) = 9.38, *p* = 0.02) and *Objects Memory* (*F*(2,77) = 10.21, *p* < 0.001) (Figure 5). Regarding *Processing Speed* (*F*(2,77) = 10.64, *p* < 0.001) and *Executive Functions* I (*χ* ^2^(2) = 14.04, *p* = 0.004), iNPH patients were more impaired than both AD and TBI groups, while AD patients were more impaired than TBI. Additionally, AD patients showed greater language impairments compared to both iNPH and TBI (*F*(2,77) = 8.77, *p* < 0.001). Full results are summarised in Table S4.

### 3.6 Cognitive Abnormalities on the Cognitron Single Scores

Individual task scores were also analysed relative to the normative dataset and compared between groups (Table S4 and Figure S2A). iNPH and AD showed widespread deficits, whereas TBI impairments were more selective, affecting tasks measuring delayed memory recognition, working memory, and language. iNPH patients were slower than AD and TBI on Trail Making (*χ* ^2^(2) = 10.96, *p* = 0.02) and performed worse than TBI on visuospatial tasks (Blocks: *F*(2,73) = 4.12, *p* = 0.04; Four Towers: *F*(2,68) = 4.04, *p* = 0.04). AD patients performed worse than both iNPH and TBI on Words Immediate (*χ* ^2^(2) = 13.90, *p* = 0.004) and Delayed (*F*(2,68) = 5.78, *p* = 0.02) Recognition, and worse than TBI on Verbal Analogies (*F*(2,70) = 4.32, *p* = 0.04). Both AD and iNPH were more impaired than TBI on Objects Immediate (*F*(2,76) = 7.21, *p* = 0.01) and Delayed (*F*(2,68) = 4.43, *p* = 0.03) Recognition, Spatial Span (*χ* ^2^(2) = 8.73, *p* = 0.02), Card Pairs (*F*(2,69) = 4.97, *p* = 0.02), Picture Completion (*χ* ^2^(2) = 9.25, *p* = 0.02), and 2D Manipulations (*F*(2,74) = 14.17, *p* < 0.001).

**FIGURE 6:**
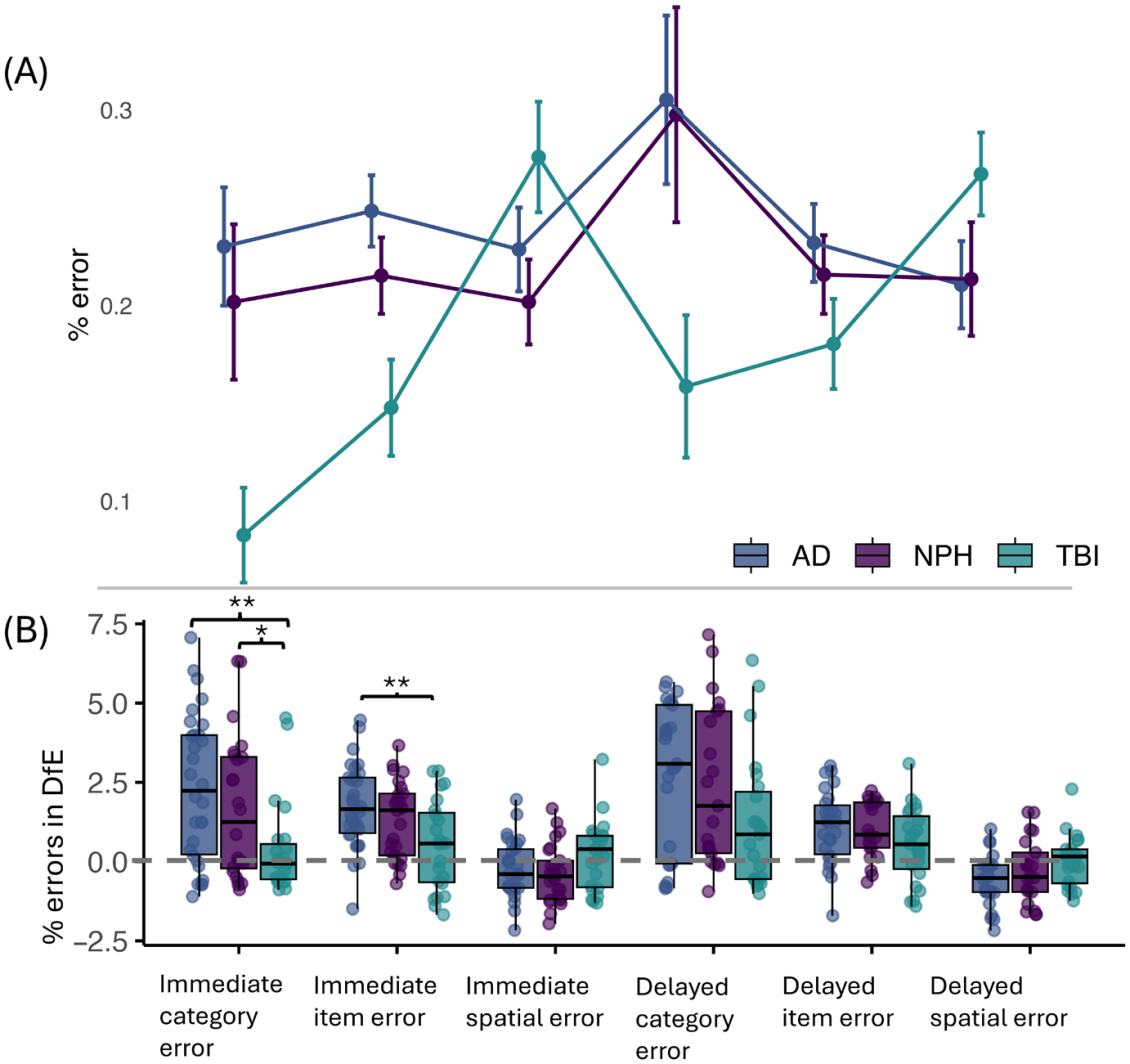
Analysis of the Objects Memory Recognition sub‐scores. (A) Between‐group comparisons of three error types derived from Objects Immediate and Delayed Recognition tasks, showing the percentage of errors in raw format. (B) Between‐group comparisons of three error types showing percentage of errors converted to DfE score. *p ≤ 0.05, **p ≤ 0.01. AD = Alzheimer's disease; iNPH = idiopathic normal pressure hydrocephalus; TBI = traumatic brain injury; DfE = Deviation from Expected.

AD patients made a significantly higher proportion of category and item errors in both Immediate (*t*(28) = 5.48, *p* < 0.001; *t*(29) = 7.23, *p* < 0.001) and Delayed (*V* = 293, *p* < 0.001; *t*(25) = 4.84, *p* < 0.001) Object Recognition compared with the normative dataset (Table S4). Similarly, relative to the normative dataset, iNPH patients made more category and item errors on both Immediate (*V* = 239, *p* = 0.01; *t*(25) = 5.29, *p* = 0.001) and Delayed (*t*(25) = 4.41, *p* < 0.001; *t*(25) = 5.02, *p* < 0.001) Object Recognition. This indicates a higher rate of low‐resolution errors and a loss of memory of fine‐grained elements of the objects (item's orientation). Accordingly, AD patients made fewer spatial errors (misremembering the item's orientation) than the normative dataset on delayed recognition trials (*t*(25) = −3.75, *p* < 0.001), but not on immediate recognition trials (*t*(29) = −1.57, *p* = 0.13), suggesting they struggled to retain details or object's identity over time. iNPH patients, instead, lost these finer details in immediate recognition (*t*(24) = −2.22, *p* = 0.04) but not in delayed recognition (*t*(22) = −1.41, *p* = 0.17). Group differences were also observed in immediate category (*χ* ^2^(2) = 8.97, *p* = 0.02) and item errors (*F*(2,75) = 5.72, *p* = 0.02), with AD and iNPH making more errors than TBI (Figure 6).

Differences on the RT scores are shown in Table S5 and Figure S2B. The sensitivity analysis of below‐chance performance is presented in Supporting Information A; Tables S6 and S7.

### 3.7 Recommendations for a Core Battery of Tasks

**FIGURE 7:**
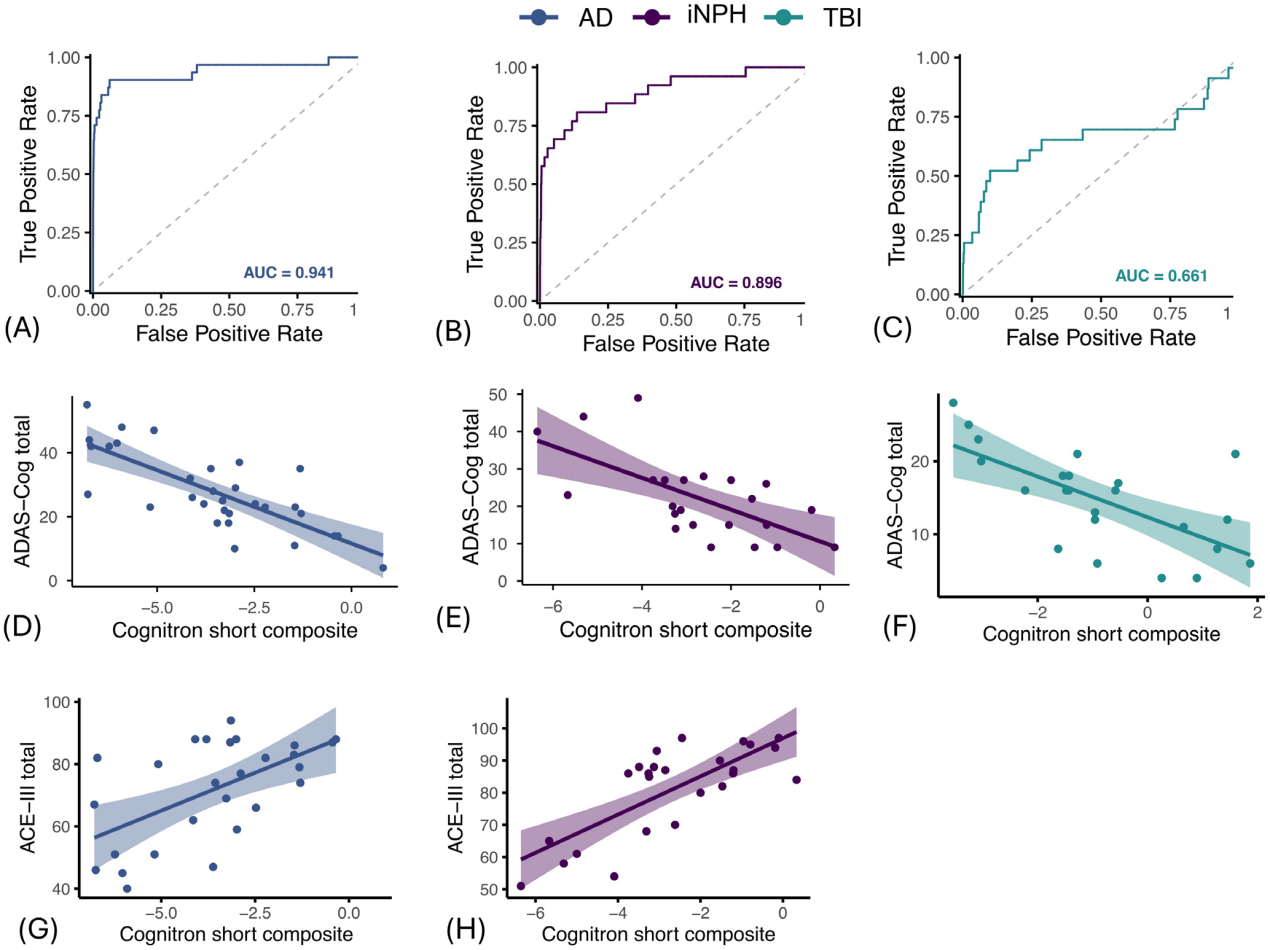
Properties of the short Cognitron composite. Receiver operating characteristic curves for the short Cognitron composite distinguishing AAD (A), iNPH (B), and TBI (C) from age‐matched controls in the normative dataset. Correlations between the Cognitron composite and the ADAS‐Cog in the AD (D), iNPH (E), and TBI (F) groups performed with linear regression. Correlations with the ACE‐III total score in the AD (G) and iNPH (H) groups. AD = Alzheimer's disease; iNPH = idiopathic normal pressure hydrocephalus; TBI = traumatic brain injury; ACE = Addenbrooke's Cognitive Examination‐III; ADAS‐Cog = Alzheimer's Disease Assessment Scale ‐ Cognitive Subscale.

We recommend a brief battery suitable across neurological conditions while minimising burden. This includes Objects and Words Recognition (immediate and delayed), Target Detection, Spatial Span, and 2D Manipulations. It takes approximately 15 min and has extensive normative data (*N* = 48,000–367,000). The Cognitron short composite explained 45.55% of the variance and demonstrated excellent accuracy for discriminating AD (AUC = 0.94) from age‐matched controls and very good accuracy for iNPH (AUC = 0.90) (Figure 7A,B). Discrimination of TBI was weaker (AUC = 0.66), although still above chance overall (Figure 7C). This pattern is consistent with the greater heterogeneity and smaller cognitive abnormalities observed in the TBI group.

The Cognitron short composite predicted ADAS‐Cog in AD (*β* = −0.77, *p* < 0.001), iNPH (*β* = −0.68, *p* < 0.001), and TBI (*β* = −0.67, *p* = 0.001), and ACE‐III in AD (*β* = 0.62, *p* = 0.002) and iNPH (*β* = 0.74, *p* < 0.001) (Figure 7D–H). Across groups, it strongly correlated with ACE‐III (*β* = 0.76, *p* < 0.001) and ADAS‐Cog (*β* = −0.77, *p* < 0.001).

### 3.8 Case Series

**FIGURE 8:**
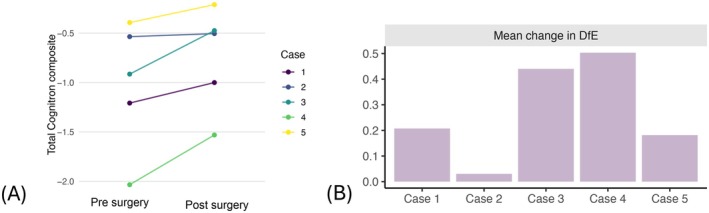
Change in cognitive performance following shunt surgery in five iNPH patients. (A) Total composite scores pre‐ and post‐shunt were computed by averaging the Deviation from Expected (DfE) score of all Cognitron tasks at each timepoint. (B) Mean change was calculated by averaging the delta values (post–pre) of all DfE‐formatted summary scores. Positive change reflects improvement in performance.

Seven iNPH patients underwent shunting during the study period and were invited to repeat the Cognitron battery 3–6 months later (Figure 8). One declined and one was medically unable to participate, leaving five postoperative cases. This pilot aimed to explore whether Cognitron scores reflect clinical change; a larger, systematic evaluation is planned.

#### 3.8.1 Case 1

A 75‐year‐old man with a 2‐year history of gait disturbance, falls, and forgetfulness (ACE‐III = 86) showed ventriculomegaly, widening of the Sylvian fissure, and temporal and hippocampal atrophy. His gait improved 17% after a tap test, and AD biomarkers were negative. One month after shunting, he reported improved alertness, bladder control, and independence, but these benefits faded by 6 months, prompting neurosurgical review for valve adjustment. Cognitron change at 6 months was minimal (+0.20 DfE).

#### 3.8.2 Case 2

A 68‐year‐old man with 2 years of gait disturbance and mild cognitive symptoms had ventriculomegaly with sulcal effacement on CT and a 20% tap test gait improvement. AD biomarkers were negative. Four months after shunting, he reported minimal gait and continence improvement; Cognitron scores showed almost no change (+0.03 DfE).

#### 3.8.3 Case 3

A 71‐year‐old retired general practitioner presented with gait apraxia, falls, and urinary urgency (ACE‐III = 82). AD CSF biomarkers were negative and MRI findings were consistent with iNPH. Tap test improvement was 7%. Four months post‐shunt, he was able to walk independently and incontinence resolved completely. Cognitron scores improved (+0.44 DfE).

#### 3.8.4 Case 4

A 75‐year‐old man with gait disturbance and urinary urgency (ACE‐III = 74) showed iNPH features with small‐vessel disease. CSF biomarkers were not suggestive of AD, though total‐tau level was mildly raised. His walk time improved by 26% after tap test. Post‐shunt, he reported gait improvement and resolution of incontinence; his wife noted faster thinking and greater independence. Cognitron change was +0.50 DfE.

#### 3.8.5 Case 5

An 80‐year‐old man had a 3‐year history of gait apraxia and urinary urgency. ACE‐III was 87, and MRI showed ventriculomegaly with disproportionately enlarged subarachnoid space features. AD biomarkers were negative and taptest improvement was modest (8%). After shunting, he regained independent ambulation and reported cognitive and continence benefits. Cognitron scores improved by +0.18 DfE.

## 4 Discussion

We demonstrated the feasibility of online cognitive testing across three burdensome conditions, where precise assessment and longitudinal monitoring are needed. The tasks showed strong discriminative power and alignment with established measures. They are broadly device‐compatible and employ an advanced normative methodology that adjusts for demographic factors, helping to overcome key limitations of traditional tests not validated across diverse cultural and linguistic contexts factors that can confound patient performance.

The overall uptake in individuals with different forms of dementia was 70%, matching other studies of digital cognitive batteries deployed in older adults both with and without clinical conditions [4, 33, 38]. The mean ADAS‐Cog and ACE‐III scores met the established dementia cut‐offs [39, 40]. The number of participants who started but did not complete the assessment was comparable to that observed when assessing young healthy adults [32], with the highest rate observed at the third difficulty level of the tasks, which requires more complex reasoning and planning. While these results demonstrate the feasibility of online testing in these populations, caution is warranted: individuals with the lowest computer literacy or most severe impairments may have been excluded. Engagement could be improved through scheduled rest breaks, tailored instruction videos, and simplified interfaces. Automatic performance thresholds (e.g., repeated errors) may also help signal when impairment is too severe for valid task engagement, prompting auto‐skipping of tasks or session termination. Alternatively, equivalent batteries of varying complexity could be deployed according to an individual's baseline cognition.

The tasks correlated strongly with the ADAS‐Cog, an outcome measure widely used in AD trials. These findings are encouraging, suggesting that online assessments could serve as valid and sensitive complements to traditional assessments in trials. Clinical trials are often more effective in early‐stage dementia, when symptoms are milder and more variable [41]. This variability typically requires larger samples to maintain statistical power, increasing costs and resources [42]. Online cognitive assessment may improve precision and sensitivity, potentially reducing the sample size needed. The association between the Cognitron tasks and the ACE‐III scores indicates strong construct validity and supports their potential for routine clinical use with the added benefits of adaptable difficulty levels, andremote longitudinal administration with greater stimuli variability. The tasks also correlated with gait measures in iNPH. Because iNPH symptoms often improve slowly over months after CSF drainage and can relapse, ongoing monitoring is critical [43]. Gait measures can detect changes after surgical intervention but can be impractical to collect frequently [15, 18]. By contrast, the Cognitron tasks can offer an accessible proxy of functional outcomes.

Identifying tasks sensitive to iNPH‐related symptoms has important clinical implications. A recent double‐blind, randomised, placebo‐controlled trial of shunting showed significant improvement in gait velocity in the treatment arm, confirming the potential benefits [15]. However, no differences emerged in cognitive outcomes, highlighting the need for more sensitive tools. Our case series provides a preliminary illustration of how performance on the tasks can mirror reports from the patients or their caregivers, though objective post‐surgery cognitive evaluation was unavailable. Moreover, subjective improvement ratings may be biased, particularly in the early postoperative period, and a selection bias toward patients who improved cannot be excluded. Repeated testing in a larger sample would confirm whether improvements are common and persistent.

Condition‐specific cognitive profiles emerged. The iNPH and AD groups exhibited pronounced global deficits relative to the population dataset, substantially exceeding those observed in the TBI group. AD patients demonstrated greater impairments in object memory, verbal memory and language, consistent with its classical amnestic and aphasic features [44]. In contrast, executive functions and processing speed deficits were most severe in the iNPH patients, in line with the fronto–subcortical profile previously reported in the disease [12, 13]. The design of the Objects Memory Recognition task allowed more detailed sub‐analyses. iNPH and AD participants made significantly more low‐resolution errors than the normative dataset and the TBI patients, misremembering both the target item and its semantic category. Interestingly, relative to the normative dataset, AD patients showed a higher proportion of fine‐grained spatial errors (i.e., misremembering the object's position/orientation) on delayed recognition trials, whereas iNPH patients made more of these errors on immediate recognition trials. iNPH memory impairments may reflect deficits in the initial encoding and attentional focus, rather pure amnesia. Accordingly, individuals with executive function impairments typically struggle more with short‐term retrieval than those with intact executive functions [45, 46]. It is however important to acknowledge that this effect did not remain significant after excluding participants who performed at or below chance, who may be driving the result. Notably, this task has shown sensitivity to dementia biomarkers such as hippocampal atrophy and amyloid positivity in undiagnosed older adults [4] but shows little learning curve with repeat deployment [47], meaning it may have relevance and accessibility for monitoring across prodromal and clinical stages.

TBI participants showed deficits in memory recognition, working memory, and language, and slower performance on Card Pairs and Stroop. We previously validated Cognitron tasks in adults with TBI and now extended this work to older individuals [27], where age‐related neurodegenerative processes may interact with TBI sequelae. However, impairments in TBI were not greater than those observed in AD or iNPH, which may reflect the non‐progressive nature of TBI and potential recovery by the subacute stage. Future studies that match baseline cognitive severity across groups will be important for isolating disorder‐specific cognitive profiles.

It should be noted that participants were recruited from both specialised memory clinics and GP surgeries across west London, a highly culturally diverse area, which broadens the demographic representativeness of our sample and improves generalisability across different levels of disease severity and healthcare settings. TBI patients were additionally recruited from St Mary's Hospital, a tertiary referral centre receiving patients from across the UK. Nevertheless, larger multicentre studies are needed to improve the generalisability and reliability of these findings.

Our study has limitations. Online administration inherently limits control over testing conditions such as caregiver interference, lack of compliance with the task rules or disengagement, which represent sources of environmental confounding factors that cannot be ruled out. We mitigated this by instructing caregivers to provide minimal assistance and ensuring tests were taken in a quiet environment and excluding data with extreme RT. However, our study sample is small and future studies should implement advanced quality assurance methods such as assessment of response variability and performance consistency at the subject level, or separate logs for caregivers to record any interruptions or assistance provided during the session. Moreover, data may not be missing at random since participants could not bypass tasks. However, allowing tasks to be skipped could still introduce bias by favouring individuals confident enough to attempt all tasks. Nine patients lacked a suitable testing device, underscoring a practical barrier to remote testing in older adults; this limitation is expected to resolve with increasing technology availability [48]. We did not characterise disease trajectories, something we plan to study in the future. Finally, the normative dataset may be influenced by recruitment bias, as online studies often over‐represent individuals with higher education, socioeconomic status, social engagement, and technological familiarity, factors linked to stronger cognitive performance [49]. Nevertheless, with almost half a million volunteers spanning different regions, age groups, and socioeconomic levels, the dataset still offers broad variability rarely achieved in population studies.

This study shows that online cognitive testing is feasible in older adults, aligns closely with gold‐standard assessments, and discriminates between different causes of cognitive decline. A 15‐min battery can be delivered remotely across conditions and disease stages, supported by a large normative dataset to control for demographic and device‐related variability, offering a cost‐effective option that reduces burden on patients and healthcare systems.

## Author Contributions

Conceptualisation: Paresh A. Malhotra, Adam Hampshire, Martina Del Giovane, Christopher Carswell, and David J. Sharp. Recruitment and data collection: Martina Del Giovane, Michael C. B. David, Magdalena A. Kolanko, Harmeena Kaur, and Christopher Carswell. Methodology: Martina Del Giovane, Adam Hampshire, and Paresh A. Malhotra. Software development: William R. Trender, and Peter J. Hellyer. Data analysis and visualisation: Martina Del Giovane and Valentina Giunchiglia. Interpretation of results: Adam Hampshire, Paresh A. Malhotra, Christopher Carswell, and Martina Del Giovane. Supervision of the study: Paresh A. Malhotra, Adam Hampshire, David J. Sharp, and Christopher Carswell. Funding acquisition: David J. Sharp and Adam Hampshire. Writing original draft: Martina Del Giovane. All authors provided revision of the manuscript.

## Funding

This work was supported by Imperial College Biomedical Research Centre, Alzheimer's Society (608 AS‐CTF‐22‐013), Medical Research Council (MR/W00710X/1, MR/W016095/1, MR/W030098/1), UK Dementia Research Institute (UKDRI‐7201).

## Conflicts of Interest

A.H. is owner/director of H2 Cognitive Designs Ltd. and Future Cognition Ltd., which produce online assessment technology and provide online survey data collection for third parties. W.R.T. is an employee of H2 Cognitive Designs LTD. P.J.H. is co‐director and co‐founder of H2 Cognitive Designs Ltd. C.C. is Chair of Association of British Neurologists NPH Specialist Interest Group, board member of the International Hydrocephalus Society, and funding trustee of South of England Neurosciences Association. D.J.S. has received funding from the Medical Research Council, National Institute of Health Research, Alzheimer's Society, Football Association, Rugby Football Union, and Premier League Rugby. P.A.M. reports funding from the National Institute for Health and Care Research (NIHR), Alzheimer's Research UK (ARUK), Fédération Internationale de Football Association (FIFA), the Football Association (FA), LifeArc, Dementias Platform UK, the Medical Research Council (MRC), and the UK Dementia Research Institute (UK DRI). He also participates in an independent Data Safety Monitoring Board for Johnson & Johnson, is a Trustee of the Alzheimer's Society, and is the NIHR Research Delivery Network (RDN) National Specialty Lead for Dementia and Neurodegeneration. All other authors have nothing to declare.

## Supporting information


**Table S1:** Description of the available Cognitron tasks.
**Table S2:** Participants' demographics.
**Table S3:** Summary of outliers removed from response time data.
**Table S4:** Comparisons of sumamry scores in deviation from expected format relative to the normative dataset within each group and between‐group comparisons.
**Table S5:** Comparisons response time scores in deviation from expected format relative to the normative dataset within each group and between‐group comparisons.
**Supporting Information A:** Analysis of below chance performance.
**Table S6:** Participants performing at or below chance level for each cognitive task.
**Table S7:** Between‐group comparisons of cognitive performance following exclusion of participants who performed at or below chance.
**Figure S1:** Results of the principal component analysis applied to the Cognitron tasks. (A) Rotated component matrix showing the loadings of each task on the extracted components. The component to which each task was assigned for the computation of composite scores is indicated by the loading shown in bold. (B) Scree plot showing the eigenvalues extracted from the principal components analysis.
**Figure S2:** Between‐group comparisons of summary measures (A) and reaction time (B) scores in Deviation from Expected (DfE) format. The dotted line represents the expected performance for an individual with the same demographic characteristics as the patient but no clinical diagnosis. *p ≤ 0.05, **p ≤ 0.01, ***p ≤ 0.001 (FDR adjusted).

## Data Availability

The data that support the findings of this study are available from the corresponding author upon reasonable request. The data are not publicly available due to privacy or ethical restrictions.

## Appendix A

**Acknowledgement list for UK Dementia Research Institute (UK DRI) Care Research & Technology (CR&T) Centre publications using the MINDER core data set**

The UK DRI CR&T Senior Management Team have agreed the list below for standardised acknowledgement. All papers should include this full list.

**Leadership and Management Infrastructure:**

Centre Director: Professor David Sharp

Centre and Research Commercialisation Manager: Danielle Wilson

Centre and Research Commercialisation Manager (interim): Melanie Bradnam

Health and Social Care Lead: Sarah Daniels

Clinical Lead: Professor Ramin Nilforooshan

General Practice Lead: Dr. David Wingfield

Human‐Centred Design Lead: Matthew Harrison

Movement Data and Living Lab Lead: Dr. Shlomi Haar

Data Science Lead: Nora Joby

Software and Engineering Lead: Anna Joffe

Scientific Project Manager: Dr. Mara Golemme

Scientific Project Manager: Dr. Stephanie Lietz

Scientific Project Manager Dr. Margherita Tecilla

**Group Leaders:** Professor David Sharp, Professor Payam Barnaghi, Professor Paul Freemont, Professor Ravi Vaidyanathan, Professor Tim Constandinou, Dr. Gregory Scott, Professor Paresh Malhotra, Imperial College London

Professor Derk‐Jan Dijk, University of Surrey, Guildford, United Kingdom

**Emerging Leaders:** Dr. Shlomi Haar and Dr. Peter Lally, Imperial College London

Dr. Valeria Jaramillo, University of Surry, Guilford, United Kingdom

**Affiliate Members:** Dame Professor Louise Robinson, Dr. Pete Lally, Dr. Chris Carswell, Dr. Bing Li, Dr. Yen Tai

**Groups: Behaviour and Cognition led by Prof David Sharp**

Affiliation: Imperial College London, Brain Sciences

Michael David

Martina Del Giovane

Neil Graham

Youssef Hbid

Julian Jeyasingh Jacob

Magdalena Kolanko

Helen Lai

Lucia M. Li

Emma Jane Mallas

Anastasia Mirza‐Davies

Abidemi Otaiku

Thomas Parker

Alina‐Irina Serban

Eyal Soreq

**Bioelectronic Systems led by Professor Timothy Constandinou**

Affiliation: Imperial College London, Electronic Engineering

Alan Bannon

Ziwei Chen

Harry Davies

Michael Drury

Cosima Graef

Charalambos Hadjipanayi

Mei Miyazaki Kirby

Alena Kutuzova

Danilo Mandic

Federico Nardi

Amir Nassibi

Nicholas Calvo Piero

Adrien Rapeaux

Assaf Touboul

Maowen Yin

Niro Yogendran

**Robotics and AI interfaces led by Professor Ravi Vaidyanathan**

Affiliation: Imperial College London, Mechanical Engineering

Rafael Calvo

Carlos Sebastian Castillo

Marco Da Re

Julio Amador Diaz Lopez

Maria Lima

Sebastian Mancero

Alina‐Irina Serban

**Machine intelligence led by Professor Payam Barnaghi—as per PB**

Affiliation: Imperial College London, Brain Sciences

Iona Biggart

Nathalia Cespedez

Nan Fletcher‐Lloyd

Antigone Fogel

Zeinab Ghannam

Samaneh Kouchaki

Chloe Walsh

**Point of care Diagnostics led by Professor Paul Freemont**

Affiliation: Imperial College London, Infectious Disease

Thomas Adam

Rory Cave

Michael Crone

Kirsten Jensen

Raphaella Jackson

Martin Tran

Alexander Webb

David Wingfield

**Sleep and Circadian led by Professor Derk Jan Dijk**

Affiliation: University of Surrey, The Surrey Sleep Research Centre as part of the School of Biosciences

Ullrich Bartsch

Ciro Della Monica

Hana Hassanin

Aravind Kumar Kamaraj

Marta Pineda Messina

Kiran K. G. Ravindran

Victoria L. Revell

Anne C. Skeldon

Kevin Wells

**Brain and movement led by Dr Shlomi Haar**

Affiliation: Imperial College London, Brain Sciences and University of Surrey, School of Psychology

Emma Burroughs

Nicolas Calvo Peiro

Sean Carr

Cosima Graef

Alena Kutuzova

Federico Nardi

Uri Rosenblum‐Belzer

Nathan Steadman

Assaf Touboul

Jenna Yun

**Groups: Computational Neurology led by Dr Gregory Scott**

Affiliation: Imperial College London, Brain Sciences

Stephen Auger

Anastasia Gailly de Taurines

Nina Moutonnet

**Helix Centre: Human Centred Design led by Matthew Harrison**

Affiliation: Imperial College London, Helix Centre and The Royal College of Art

Sophie Horrocks

Tori Simpson

**Software engineering team led by Anna Joffe**

Affiliation: Imperial College London, Brain SciencesRamsheed Abdul Rahim

Amer Marzuki

Mark Woodbridge

**Data Science team led by Nora Joby**

Affiliation: Imperial College London, Brain ScienceEthan de Villiers

Gaia Frigerio

**Site Investigators and Key Personnel:**

**Name and Affiliation: Surrey and Borders Partnership NHS Foundation Trust (Site and Sponsor)**

Chief Investigator: Professor Ramin Nilforooshan

Research and Development Managers: Jessica True

Research Co‐ordinator: Chloe Walsh

Clinical Monitoring Team: Nicole Whitethread, Matthew Purnell, Vaiva Zarombaite, Lucy Copps, Olivia Knight, Gaganpreet Bangar, Sumit Dey, Chelsea Mukonda, Jessica Hine, Luke Mallon, Saijal Jhala, Oliver Sargentoni, Amy Alves, Annabel Connor, Josie Jenkinson

**Name and Affiliation: Hammersmith and Fulham Partnership**

Principal Investigator: Dr. David Wingfield

Research Nurse/Paramedic: Monica Morim

Clinical Studies Officers/Research Technicians: Success Fabusoro, Gaia Frigerio, Maria Rasulo, Catalina Chavarro Novoa, Martynas Stonkus, Prital Patel, Zara Prem

**Name: Minder Digitally Enabled Care for Dementia (MinderCare)**

**Imperial College Healthcare NHS Trust, Imperial College London**

Principal Investigator: Professor David Sharp

Co‐Investigators (not listed elsewhere): James Bird, Sarah Pearse, Aglaja Dar, Pandora Wright, David Wingfield

Programme Lead: Sarah Daniels

Scientific Project Manager: Margherita Tecilla

MinderCare Clinical Team: Joanna James, Janibo Amade Cassimo, Lucia Li, Anastasia Mirza‐Davies, Julian Jeyasingh Jacobs, Zinca Zecevic

Research Technicians: Success Fabusoro, Gaia Frigerio, Maria Rasulo, Catalina Chavarro Novoa

Imperial Clinical Trials Unit: Suzie Cro, Martin Orr

**Name: Howz**

Co‐founder and Chief Technology Officer: Jonathan Burr

Managing Director: Kate Fairhurst

Chief Clinical Officer: Louise Rogerson

Data lead: Oliver Dickson
