# Supplementary material for: Digital Cognitive Phenotyping for Differential Diagnosis and Monitoring in Neurological Conditions
Source: Ann Clin Transl Neurol. 2026 Jul 11:10.1002/acn3.70451. Online ahead of print. doi: 10.1002/acn3.70451 (PMC13394833; doi:10.1002/acn3.70451)
Supplement: Supplementary file 1 — Table S1: Description of the available Cognitron tasks. Table S2: Participants' demographics. Table S3: Summary of outliers removed from response time data. Table S4: Comparisons of sumamry scores in deviation from expected format relative to the normative dataset within each group and between‐group comparisons. Table S5: Comparisons response time scores in deviation from expected format relative to the normative dataset within each group and between‐group comparisons. Supporting Information A: Analysis of below chance performance. Table S6: Participants performing at or below chance level for each cognitive task. Table S7: Between‐group comparisons of cognitive performance following exclusion of participants who performed at or below chance. Figure S1: Results of the principal component analysis applied to the Cognitron tasks. (A) Rotated component matrix showing the loadings of each task on the extracted components. The component to which each task was assigned for the computation of composite scores is indicated by the loading shown in bold. (B) Scree plot showing the eigenvalues extracted from the principal components analysis. Figure S2: Between‐group comparisons of summary measures (A) and reaction time (B) scores in Deviation from Expected (DfE) format. The dotted line represents the expected performance for an individual with the same demographic characteristics as the patient but no clinical diagnosis. *p ≤ 0.05, **p ≤ 0.01, ***p ≤ 0.001 (FDR adjusted). [file ACN3-9999-0-s001.pdf]

Supplementary table 1. Description of the available Cognitron tasks.

Tasks	Difficulty	Study		Paradigm	End criteria	Summary Score	Additional scores	Mean completion time in secs (SD)	Norms available	Norms for age > 60
Motor Control	1	A, B, C	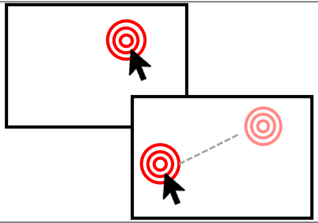	Participants are shown a red target appearing at different locations of the screen and have to tap on it as quickly as possible.	30 trials	Median RT	Mean distance from the target	46.64 (59.29)	28183	2971
Words Immediate and Delayed Recognition	1	C	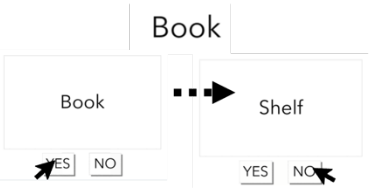	Participants are shown a list of words. Afterwards, they are asked to select the words they have seen within a list containing confounders. This task is repeated at the end of the battery to measure delayed memory recognition.	12 words	Total correct	Median RT for correctly recognised words	IR: 77.78 (26.10); DR:63.91 (21.78)	IR:100871; DR:99955	IR:14615; DR:14286
Target Detection	1	C	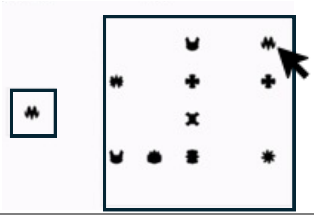	Participants must identify and click on all the stimuli in the right hand-side panel that match the target showed on the left hand-side panel.	120 targets appearing on the screen	Total correct	Median RT for correctly identified targets	123.16 (0.26)	367295	59265
Emotional Discrimination	1	A, C	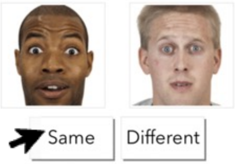	Participants are shown pairs of faces and have to indicate whether these show the same emotional expression.	50 pairs of faces	Total correct	Median RT	216.18 (83.59)	366397	59229
2D Manipulations	1	A, B, C	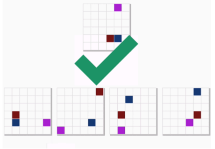	Participants must identify a target array of objects in a choice of different arrays. The target will have been rotated through either 90, 180 or 270 degrees.	3 minutes	Total correct	Median RT for correct answers	180.84 (1.15)	367515	59332

Digit Span	2	A, B, C	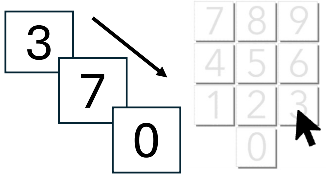	Participants are asked to memorise a list of digits and then replicate it. The list of digits increments in length every correct trial.	3 consecutive failures	Total correct	Median RT	195.96 (83.66)	366034	58658
Spatial Span	2	A, B, C	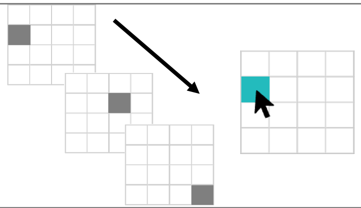	Participants are asked to memorise a sequence of grey square appearing at different location of a 4X4 grid. The number of squares increments in length every correct trial. This is a variant of the classic Corsi Block Tapping paradigm (67)	3 consecutive failures	Total correct	Median RT	125.03 (42.21)	367265	59198
Blocks	3	A, B, C	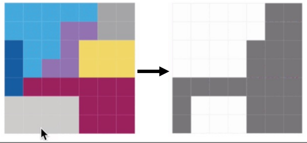	Participants are asked to remove blocks of different colours and shapes from one array to match the disposition of a target array.	15 trials	Total correct	Median RT	269.23 (143.98)	367624	59376
Tower of London	3	A, C	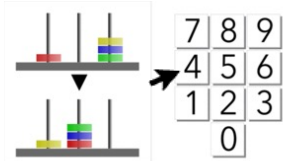	This is a problem-solving task measuring executive functions (Shallice, 1982). Participants are given one starting board with coloured pegs and are asked to count the sequence of moves needed to reorder the pegs so that they match a target disposition.	10 trials	Total correct	Median RT for correct answers	321.41 (446.29)	367975	59430
Verbal Analogies	3	C	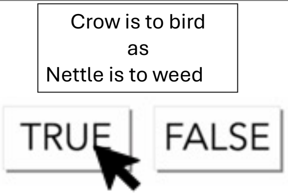	Participants are shown verbal analogies (e.g. dog is to animal as daisy if to flower) and must indicate whether these are correct or not.	3 minutes	Total correct	Median RT	180.35 (0.31)	367611	59373

Word Definitions	3	B, C	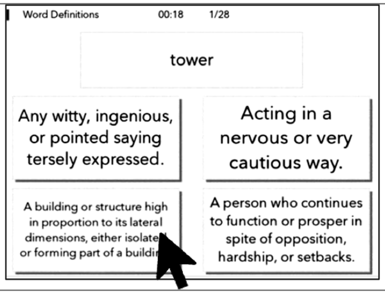	Participants are shown a word and 4 possible definitions and will have to tap on the correct definition within a maximum amount of time.	28 trials, 20 seconds per trial	Total correct	Median RT	255.40 (79.65)	367607	59372
Objects Immediate and Delayed Recognition	1	B, C	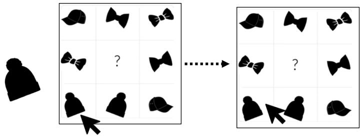	Participants are shown a sequence of target objects. Afterwards, they are asked to identify these targets in different arrays of objects. The arrays contain identical but mirrored version of the target, an object from the same semantic category of the target and 4 objects from different semantic categories. This task is repeated at the end of the battery to measure delayed memory recognition.	20 objects	Total correct (correct category, item and orientation)	Time to complete task, category error (wrong category, item and orientation, item error (correct category but wrong item and orientation, orientation error (correct category and item but wrong orientation)	IR:241.36 (113.32); DR:172.02 (171.56)	IR:50081; DR:48070	IR:8494; DR:8000
Simple RT	1	A, B, C	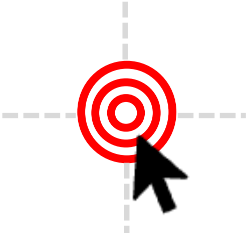	Participants must respond as quickly as they can to a red target that appears on the screen at different timepoints.	60 trials	Mean RT	SD of RT	112.12 (10.19)	3874	990

Choice RT	1	A, B, C	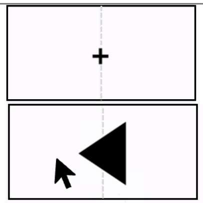	Participants are shown an arrow pointing either left of right and have to respond accordingly to it tapping/clicking on the left or right-hand side of the screen.	60 trials	Mean RT	SD of RT	123.45 (7.89)	4237	983
Trail Making	1	A, C	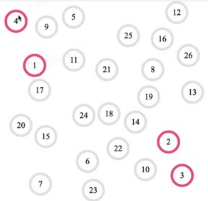	This task is the digital readaptation of the classic paper-based Trail Making test (69). Participants have to link circled numbers in numeric order in part A, and circled numbers and letters in alternating order in part B.	Completion of array B	Completion of array A	Mean RT B, mean RT A	266.49 (182.21)	6526	1752
Pairs Associate Learning	2	A, C	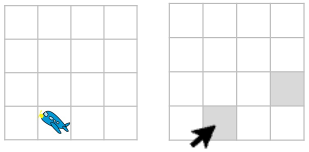	Some images are displayed at random locations in a 4x4 grid. Once all images have been revealed and hidden, each object is displayed on the left-hand side panel. Individuals must select the location each object appeared at. If the participant answers correctly, the next trial will have one additional object.	3 consecutive failures	Total correct	Median RT	134.02 (66.19)	3144	875
Switching Stroop	3	A, B, C	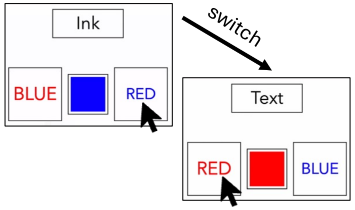	Participants have to indicate the colour of a tile by tapping on one of two words: "blue" and "red," which are coloured either blue or red. A box will indicate which modality they will have to provide the answer in (i.e., the colour or text of the word).	60 trials	Total correct	Median RT	340.98 (132.24)	5836	1595
Picture Completion	2	A, C	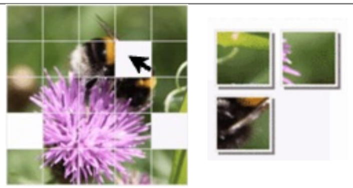	Participants must correctly assign missing pieces to the empty spaces of a picture. The pieces are sometimes rotated through 90, 180 or 270 degrees.	12 pictures	Total errors	Time taken to complete task	635.27 (385.20)	2222	681

Card Pairs	2	A, C	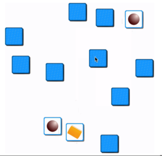	Participants are shown an array of pairs of identical cards. These cards are then placed face down and participants must remember and identify the location of the identical pairs of cards.	6 trials	percentage correct answers	Time taken to complete task	352.98 (294.76)	5801	1594
Four Towers	3	C	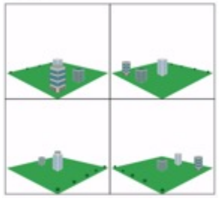	Participants are shown four tridimensional rotations of the same space and will have to indicate which one is incorrect.	12 trials	Total correct	Median RT	250.49 (177.19)	106083	15593

1. Responses based on simple retainable rule 2. Responses require retaining information being retained in working memory. 3. Responses require more complex reasoning/planning strategies. IR=immediate recognition. DR=delayed recognition

Supplementary table 2. Participants' demographics.

	**iNPH**	**AD**	**TBI**	**Group differences**
	26	31	23	
**Mean age (SD)**	72.04 (0.64)	74.26 (8.71)	69.87 (9.45)	$F_{(2,77)}$=1.85, p=0.17*
**Sex, N Male**	18	20	15	$\chi^2_{(2)}$=0.16, p=0.93†
**Education (N)**				$\chi^2_{(6)}$=4.19, p=0.65†
Pre-GCSE	3	4		
High School	10	12	10	
Degree	13	14	12	
PhD		1	1	
**Device**				$\chi^2_{(10)}$=9.77, p=0.46†
Android Phone			2	
Android tablet		2		
Windows computer	14	18	12	
Apple phone	2	2		
Apple tablet	5	4	3	
Apple computer	5	5	6	
**Ethnicity**				$\chi^2_{(6)}$=4.45, p=0.62†
Asian or Asian British	2	1	3	
Black, black British, Caribbean or African	2		1	
White	21	29	18	
Mixed or multiple ethnic groups	1	1	1	
**Language N English**	25	31	20	$\chi^2_{(2)}$=4.84, p=0.09†
**Handedness N (%) right**	21	27	20	$\chi^2_{(4)}$=3.57 p=0.47†
**ACE-III total mean (SD)**	81.12 (14.10)	71.85 (16.29)		W=458.5, p=0.03‡
ACE-III Memory	19.38 (5.57)	12.56 (6.23)		$t_{(46.78)}$=-4.04, p<0.001 (AD<iNPH)§

ACE-III Attention	15.92 (3.36)	12.84 (4.15)		W=156.5, p=0.003 (AD<iNPH)‡
ACE-III Language	23.75 (2.80)	20.88 (5.40)		W=190, p=0.03(AD<iNPH) ‡
ACE-III Fluency	7.67 (3.71)	7.56 (3.59)		$t_{(46.74)}$=-0.10, p=0.92§
ACE-III Visuospatial	13.88 (3.05)	12.60 (3.74)		W=231, p=0.16‡
**ADAS-Cog mean (SD)**	22.22 (10.90)	27.90 (12.38)	14.74 (6.72)	$\chi^2_{(2)}$=17.45, p<0.001 (AD>TBI, iNPH>TBI)¶

*One-way ANOVA, † Chi[2] test, ‡ Wilcoxon-test, § Unpaired t test, ¶ Kruskal-Wallis with post-hoc Dunn test

Supplementary Table 3. Summary of outliers removed from the dataset for the response time scores.

	**TBI**	**AD**	**iNPH**
Simple RT	1	1	
Choice RT	1		
Trail Making B		4	
Motor Control	1	2	2
Words Immediate (RT)		2	1
Words Delayed (RT)		1	
Paired Associate Learning (RT)	1	4	5
Blocks (RT)		2	1
Card Pairs (time)	1	5	3
Four Towers (RT)		1	
Picture Completion (time)	1	5	1
Stroop (RT)			1
Tower of London (RT)	1		
Digit Span (RT)		1	
Spatial Span (RT)	1		
2D Manipulations (RT)		3	5

No outliers were identified for tasks which are not show in the table

Supplementary table 4. Comparisons of Deviation from Expected scores relative to the normative dataset for each clinical group and between-group comparisons

	**TBI vs norms**	**AD vs norms**	**iNPH vs norms**	**Group differences**	**Post-hoc tests**
Global Cognition	$t_{(22)}=-3.29$, $p=0.02$	$t_{(30)}=-8.57$, $p<0.001$	$t_{(25)}=-8.42$, $p<0.001$	$F_{(2,77)}=9.74$, $p<0.001$	AD<TBI***, iNPH<TBI***
Objects Memory	$t_{(22)}=-1.67$, $p=0.11$	$t_{(30)}=-7.74$, $p<0.001$	$t_{(25)}=-6.70$, $p<0.001$	$F_{(2,77)}=10.21$, $p=0.01$	AD<TBI***, iNPH<TBI**
Words Memory	t $t_{(22)}=-1.97$, $p=0.06$	$t_{(30)}=-6.61$, $p<0.001$	$t_{(25)}=-4.40$, $p<0.001$	$\chi^2_{(2)}=9.38$, $p=0.04$	AD<TBI**, iNPH<TBI*
Language	$t_{(22)}=-1.73$, $p=0.10$	$t_{(30)}=-6.37$, $p<0.001$	$t_{(25)}=-2.61$ $p=0.02$	$F_{(2,77)}=8.77$, $p<0.001$	AD<iNPH**, AD<TBI***
Processing Speed	$t_{(22)}=-1.53$, $p=0.14$	$t_{(30)}=-4.95$, $p<0.001$	$t_{(25)}=-8.32$, $p<0.001$	$F_{(2,77)}=10.64$, $p<0.001$	iNPH<AD*, iNPH<TBI***, AD<TBI**
Executive Functions I	$V=114$ $p=0.54$	$V=65$, $p<0.001$	$t_{(25)}=-6.66$, $p<0.001$	$\chi^2_{(2)}=14.04$, $p=0.01$	iNPH<AD*, iNPH<TBI***, AD<TBI*
Executive Functions II	$t_{(22)}=-2.45$, $p=0.02$	$t_{(30)}=-8.25$, $p<0.001$	$t_{(25)}=-6.22$, $p<0.001$	$F_{(2,77)}=3.79$, $p=0.05$	AD<TBI*, iNPH<TBI*
OI category error	$V=143$ $p=0.89$	$t_{(28)}=5.48$, $p<0.001$	$V=239$, $p=0.01$	$\chi^2(2)=8.97$ $p=0.02$	AD<TBI**, iNPH<TBI*
OI item error	$t_{(22)}=1.73$, $p=0.08$	$t_{(29)}=7.23$, $p<0.001$	$t_{(24)}=5.29$ $p<0.001$	$F(2,75)=5.72$ $p=0.02$	AD<TBI**
OI spatial error	$t_{(22)}=0.92$, $p=0.37$	$t_{(29)}=-1.57$, $p=0.13$	$t_{(24)}=-2.22$, $p=0.04$,	$F(2,75)=2.65$ $p=0.09$	
OD category error	$V=215$, $p=0.02$	$V=293$, $p<0.001$	$t(20)=4.41$, $p<0.001$	$\chi^2(2)=4.99$, $p=0.09$	
OD item error	$t_{(22)}=2.15$, $p=0.04$	$t_{(25)}=4.84$, $p<0.001$	$t_{(20)}=5.02$, $p<0.001$	$F(2,67)=1.81$, $p=0.18$	
OD spatial error	$t_{(22)}=0.12$, $p=0.91$	$t_{(25)}=-3.75$, $p<0.001$	$t_{(22)}=-1.41$, $p=0.17$	$F(2,67)=2.93$, $p=0.08$	
Words Immediate	$V=90$, $p=0.15$	$t_{(30)}=-7.22$, $p<0.001$	$t_{(25)}=-3.16$, $p=0.004$	$\chi^2_{(2)}=13.90$, $p=0.01$	AD<iNPH**, AD<TBI***
Words Delayed	$t_{(21)}=-1.65$, $p=0.11$	$V=34$, $p<0.001$	$t_{(21)}=-2.04$, $p=0.05$	$F_{(2,68)}=5.78$, $p=0.02$	AD<iNPH**, AD<TBI**

Objects Immediate	$t_{(22)}$=-2.22, p=0.04	$t_{(29)}$=-7.79, p<0.001	$t_{(25)}$=-5.20, p<0.001	$F_{(2,76)}$=7.21, p=0.01	AD<TBI**, iNPH<TBI**
Objects Delayed	$t_{(22)}$=-3.29, p=0.003	$t_{(25)}$=-7.33, p<0.001	$t_{(21)}$=-5.85, p<0.001	$F_{(2,68)}$=4.43, p=0.03	AD<TBI*, iNPH<TBI*
Paired Associate Learning	$t_{(22)}$=-3.42, p=0.002	$t_{(27)}$=-8.60, p<0.001	$t_{(25)}$=-5.78, p<0.001	$F_{(2,74)}$=1.86, p=0.18	
Blocks	$t_{(22)}$=-2.56, p=0.02	$t_{(30)}$=-3.89, p<0.001	$t_{(21)}$=-7.21, p<0.001	$F_{(2,73)}$=4.12, p=0.04	iNPH<TBI*
Card Pairs	$t_{(22)}$=-3.24, p=0.004	$t_{(26)}$=-7.20, p<0.001	$t_{(21)}$=-6.25, p<0.001	$F_{(2,69)}$=4.97, p=0.02	AD<TBI**, iNPH<TBI*
Four Towers	V=114, p=0.48	$t_{(25)}$=-3.67, p=0.001	$t_{(21)}$=-4.81, p<0.001	$F_{(2,68)}$=4.04, p=0.04	iNPH<TBI*
Picture Completion	V=130, p=0.82	$t_{(27)}$=-4.00, p<0.001	$t_{(21)}$=-4.11, p<0.001	$\chi^2_{(2)}$=9.25, p=0.02	AD<TBI**, iNPH<TBI**
Emotion Discrimination	$t_{(22)}$=-1.53, p=0.14	V=77, p<0.001	V=92, p=0.03	$\chi^2_{(2)}$=1.24, p=0.54	
Stroop	$t_{(22)}$=-1.31, p=0.21	$t_{(28)}$=-3.28, p=0.003	$t_{(24)}$=-4.52, p<0.001	$F_{(2,74)}$=2.57, p=0.09	
Tower of London	$t_{(21)}$=-1.25, p=0.22	$t_{(27)}$=-3.44, p=0.002	$t_{(21)}$=-4.65, p<0.001	$F_{(2,69)}$=0.96, p=0.40	
Word Definitions	V=41, p=0.004	$t_{(27)}$=-5.99, p<0.001	V=46, p=0.01	$\chi^2_{(2)}$=5.02, p=0.09	
Verbal Analogies	$t_{(22)}$=3.66, p=0.001	$t_{(27)}$=-9.09, p<0.001	$t_{(21)}$=-5.80, p<0.001	$F_{(2,70)}$=4.32, p=0.04	AD<TBI**
Digit Span	$t_{(22)}$=-2.11, p=0.05	V=31, p<0.001	$t_{(24)}$=-2.91, p=0.01	$\chi^2_{(2)}$=2.25, p=0.36	
Spatial Span	V=67, p=0.03	V=15, p<0.001	$t_{(23)}$=-5.99, p<0.001	$\chi^2_{(2)}$=8.73, p=0.02	AD<TBI**, iNPH<TBI**
2D Manipulations	$t_{(20)}$=0.00, p=1.00	$t_{(29)}$=-5.77, p<0.001	$t_{(23)}$=-8.61, p<0.001	$F_{(2,74)}$=14.17, p<0.001	AD<TBI***, iNPH<TBI***
Target Detection	$t_{(21)}$=-2.04, p=0.05	$t_{(29)}$=-3.13, p=0.004	$t_{(25)}$=-6.97, p<0.001	$F_{(2,75)}$=3.11, p=0.07	
Motor Control	$t_{(21)}$=-2.51, p=0.02	$t_{(28)}$=-3.67, p<0.001	V=19, p<0.001	$\chi^2(2)$=6.40 p=0.06	

Trail Making	$V=183$, $p=0.18$	$V=153$, $p=0.58$	$t_{(25)}=-3.85$, $p<0.001$	$\chi^2_{(2)}=10.96$, $p=0.02$	iNPH<AD*, iNPH<TBI**
Choice RT	$V=67$, $p=0.03$	$t_{(29)}=-3.73$, $p<0.001$	$t_{(23)}=-5.13$, $p<0.001$	$\chi^2(2) = 6.54$ $p=0.06$	
Simple RT	$V=67$, $p=0.05$	$V=26$, $p<0.001$	$t_{(25)}=-4.94$, $p<0.001$	$\chi^2_{(2)}=5.70$, $p=0.08$	

*p≤0.05, **p≤0.01, ***p≤0.001 (FDR adjusted). P values underwent FDR correction for multiple comparisons, OI=Objects immediate, OD=Objects delayed

Supplementary Table 5. Comparisons of the response time (RT) scores in Deviation from Expected format relative to the normative dataset within each group and between-group comparisons

	**TBI vs norms**	**AD vs norms**	**iNPH vs norms**	**Group differences**	**Post-hoc comparisons**
Words Immediate (RT)	$V=104$, $p=0.41$	$V=27$, $p<0.001$	$V=72$, $p=0.02$	$\chi^2_{(2)}=7.27$, $p=0.06$	
Words Delayed (RT)	$V=111$, $p=0.67$	$V=22$, $p<0.001$	$V=33$, $p=0.002$	$\chi^2_{(2)}=12.85$, $p=0.002$	AD<TBI***, AD<iNPH*, iNPH<TBI*
Objects Immediate (duration)	$V=116$, $p=0.63$	$V=68$, $p<0.001$	$V=47$, $p<0.001$	$\chi^2_{(2)}=4.40$, $p=0.16$	
Objects Delayed (duration)	$V=63$, $p=0.09$	$V=34$, $p<0.001$	$V=34$, $p=0.003$	$\chi^2_{(2)}=2.77$, $p=0.28$	
Paired Associate Learning (RT)	$V=80$, $p=0.21$	$V=17$, $p<0.001$	$V=11$, $p<0.001$	$\chi^2_{(2)}=8.28$, $p=0.06$	
Blocks (RT)	$V=67$, $p=0.09$	$t(28)=-1.75$, $p=0.09$	$t(20)=-3.50$, $p=0.003$	$\chi^2_{(2)}=4.04$, $p=0.17$	
Card Pairs (duration)	$V=59$, $p=0.09$	$t(21)=-5.33$, $p<0.001$	$V=1$, $p<0.001$	$\chi^2_{(2)}=6.77$, $p=0.07$	
Four Towers (RT)	$V=76$, $p=0.12$	$t(24)=-2.11$, $p=0.06$	$V=121$, $p=0.87$	$\chi^2_{(2)}=1.74$, $p=0.44$	

Picture Completion (duration)	V=25, p<0.001	t(22)=-5.37, p<0.001	V=2, p<0.001	$\chi^2_{(2)}$=4.76, p=0.16	
Stroop (RT)	V=39, p=0.02	V=70, p<0.001	t(23)=-6.01, p<0.001	$\chi^2_{(2)}$=7.32, p=0.06	
Tower of London (RT)	V=65, p=0.14	t(27)=-2.22, p=0.05	V=79, p=0.14	$\chi^2_{(2)}$=0.01, p=0.10	
Word Definitions (RT)	V=130, p=0.92	t(27)=-5.98, p<0.001	V=58, p=0.03	$\chi^2_{(2)}$=13.86, 0.02	AD<TBI***, AD<iNPH*, iNPH<TBI*
Verbal Analogies (RT)	V=67, p=0.09	t(27)=-6.10, p<0.001	t(21)=-4.06, p<0.001	$\chi^2_{(2)}$=9.78, p=0.05	AD<TBI**
Digit Span (RT)	V=90, p=0.21	V=82, p=0.001	V=19, p<0.001	$\chi^2_{(2)}$=3.14, p=0.25	
Spatial Span (RT)	t(21)=-2.25, p=0.09	V=158, p=0.09	V=32, p<0.001	$\chi^2_{(2)}$=4.35, p=0.16	
2D Manipulations (RT)	V=157, p=0.66	V=96, p=0.04	V=16, p<0.001	$\chi^2_{(2)}$=8.81, p=0.05	AD<TBI*, iNPH<TBI**
Target Detection (RT)	t(21)=-2.04, p=0.11	t(29)=-1.75, p=0.10	t(25)=-5.14, p<0.001	$F_{(2,75)}$ =2.22, p=0.16	

*p≤0.05, **p≤0.01, ***p≤0.001 (FDR adjusted). P values underwent FDR correction for multiple comparisons. RT=Response time

Supplement A. Analysis of below chance performance

Any participant scoring below the 5th percentile of this simulated distribution was classified as performing worse than 95% of random guessers under the same conditions. We excluded these participants from the dataset and then reassessed the deviation from expected (DfE) scores for each group relative to the normative dataset and repeated the between-group comparisons. Below is an overview of the approach taken to determine performance at chance for each task.

Digit Span and Spatial Span

In these tasks, participants must memorise and replicate a sequence of numbers on a keypad (Digit Span) or square locations (Spatial span) on a 4×4 grid. The sequence starts at two items and increases by one unit for each correct response. The test ends after three consecutive errors. I used R Studio 4.3.3 to generate 100,000 random guesses on each trial while following the same task rules. The maximum sequence length reached by each simulated guesser was recorded, and the 5th percentile of this distribution was used as the below-chance cutoff.

2D Manipulations

This task measures how many visual arrays participants correctly identify within 3 minutes. I first examined the minimum number of arrays correctly identified by individuals aged 55 or older in the large normative dataset. This showed that participants could complete 1 to 60 trials per minute. Each of 100,000 simulations randomly selected a number of trials from 1 to 60. It then generated responses using a binomial distribution, with a probability (p) of 1/4, as each trial has four options with only one correct answer. This process was then repeated to create a distribution of total correct scores under guessing. The 5th percentile of this distribution served as the below-chance threshold. Participants scoring below this cutoff performed worse than 95% of random guessers.

Verbal Analogies

Participants determine whether each verbal analogy is correct or incorrect, answering as many times as possible within 3 minutes. They receive +1 point for a correct answer and -1 point for an incorrect answer. To simulate guessing, each virtual participant attempted a random number of items uniformly sampled between n=-28 and 76. These values were extracted based on the minimum and maximum score achieved by individuals of the large normative dataset aged 55 or older. Each response had a 50% chance of being correct. The final score was calculated as: Final Score=2×(Correct Answers) − n. Repeating this 100,000 times generated a distribution of expected scores under random guessing. The 5th percentile of this distribution was set as the below-chance cutoff.

Word Definitions

Participants select the correct word definition from four choices across 28 trials. I modelled the total correct responses as a binomial distribution with parameters n=28 and p=0.25, given that for each of 28 trials there is a 1 in 4 chance of answering correctly. The 5th percentile of this distribution was used as the below-chance threshold.

Tower of London

This task consists of 10 trials, each with 9 possible answers. A random guesser has a 1/9 chance of selecting the correct answer per trial. The total number of correct responses follows a binomial distribution with parameters n=10 and p=0.11. The 5th percentile of this distribution was used as the below-chance threshold.

Emotion Discrimination

Participants complete 50 trials, deciding whether two faces display the same or different emotion. Each response has a 50% chance of being correct. The total number of correct responses follows a binomial distribution with parameters n=50 and p=0.5. The 5th percentile of this distribution was used as the below-chance threshold.

Words Immediate and Delayed Recognition

Participants must say whether each of 24 words was or not on a list of 12 target words. Since a guessing participant has a 50% chance of answering each item correctly, the total correct responses follow a binomial distribution with n=24 and p=0.5. The 5th percentile of this distribution was used as the below-chance threshold.

Four Towers

Participants complete 12 trials, selecting which of four tridimensional rotations of the same space is the incorrect one. They receive +1 point for a correct answer and -1 point for an incorrect answer. A random guesser has a 1 in 4 (0.25) chance of answering correctly. The total number of correct responses under guessing is $12 \times 0.25 = 3$. The total number of incorrect responses will therefore be $12 - 3 = 9$. The total score is calculated as: (correct responses under guessing) −(incorrect responses), which would be -6. Any participant scoring below -6 was classified as performing below chance.

Stroop

Participants complete 60 trials, where they have a 50% chance of selecting the correct answer. The total number of correct responses follows a binomial distribution with n=60 and p=0.50. The 5th percentile of this distribution was used as the below-chance threshold. Participants scoring 24 or fewer correct responses were classified as performing below chance.

Objects Immediate and Delayed Recognition

Participants are shown a sequence of 20 target objects and then have to identify these in an array of 8 objects. To assess chance-level performance, I focused on the category error, which occurs when participants select the object belonging to the wrong semantic category. There are 8 objects in each array, with 4 distractors and 4 targets. The chance of selecting a distractor is 4 out of 8, or 50% for each array. With 20 arrays, if participants were guessing randomly, we would expect them to make 10 errors. Participants who make more than 10 errors were considered to perform below chance.

Picture Completion, Card Pairs, Blocks, and Target Detection were too complex to derive a reliable chance-level threshold. However, no participants scored below the minimum observed score from the age-matched normative dataset.

Results

The numbers of participants identified as performing below chance for each task are shown in Supplementary table 7. Following removal of these participants from the analysis, TBI participants were no longer impaired on Word Definitions compared to the large normative dataset (V=114, p=0.48). No material changes in mean cognitive deficits relative to the normative dataset were observed for the AD group. NPH participants no longer showed a significantly higher number of spatial errors on the Objects Immediate Recognition ($t_{(23)}$=-1.69, p=0.12). However, they still made significantly more category and item errors compared to the normative dataset on both immediate (V=227, p=0.03; $t_{(23)}$=5.23, p<0.001) and delayed (V=171, p=0.002; V=178, p<0.001) recognition (Supplementary table 7).

Supplementary table 6. Participants performing at or below chance level for each cognitive task

Task	Score	AD below chance	iNPH below chance	TBI below chance
Words Immediate Recognition	Total correct	0	0	0
Emotional Discrimination	Total correct	0	0	0
2D Manipulations	Total correct in 3 minutes	0	0	0
Digit Span	Total correct	0	0	0
Spatial Span	Total correct	3	0	1
Tower of London	Total correct	0	0	0
Verbal Analogies	Total correct in 3 minutes	0	0	0
Word Definitions	Total correct	1	0	0
Words Delayed Recognition	Total correct	0	0	0
Objects Immediate Recognition	Total correct	1	3	0
Pairs Associate Learning	Total correct	0	0	0
Switching Stroop	Total correct	0	0	0
Four Towers	Total correct	4	7	2
Objects Delayed Recognition	Total correct	2	3	1

Supplementary table 7. Between-group comparisons of cognitive performance following exclusion of participants who performed below chance

	TBI	p (FDR)	AD	iNPH	Group differences	Post-hoc
Objects Immediate	$t_{(22)}=-2.22$, $p=0.037$	0.08	$t_{(28)}=-7.53$, $p<0.001$	$t_{(22)}=-4.69$, $p<0.001$	$F_{(2,72)}=7.18$, $p<0.001$	AD<TBI***

Objects Delayed	$t_{(21)}=-3.02$, $p=0.007$	0.02	$V=10$ $p<0.001$	$t_{(28)}=-5.48$, $p<0.001$	$F_{(2,64)}=7.54$, $p<0.001$	AD<TBI *
Spatial Span	$t_{(21)}=-2.22$, $p=0.038$	0.08	$V=15$ $p<0.001$	$t_{(23)}=-5.99$, $p<0.001$	$\chi^2_{(2)}=11.13$, $p<0.001$	AD<TBI***, NPH<TBI **
Four Towers	$V=41$ $p=0.004$	0.02	$t_{(26)}=-5.90$, $p<0.001$	$V=46$ $p=0.007$	$F_{(2,67)}=4.06$, $p=0.02$	NPH<TBI *
Word Definitions	$V=114$ $p=0.48$	0.59	$t_{(24)}=-3.40$, $p=0.002$	$t_{(21)}=-4.81$, $p<0.001$	$\chi^2_{(2)}=4.17$, $p=0.12$	AD<TBI **
OI category error	$V=143$, $p=0.89$	0.89	$t_{(28)}=5.33$, $p<0.001$	$V=203$, $p=0.048$	$\chi^2_{(2)}=8.62$, $p=0.01$	AD>TBI * iNPH>TBI *
OI item error	$t_{(22)}=1.73$, $p=0.10$	0.15	$t_{(28)}=7.27$, $p<0.001$	$t_{(22)}=4.96$, $p<0.001$	$F_{(2,72)}=5.98$, $p<0.001$	AD<TBI**
OI spatial error	$t_{(22)}=0.92$, $p=0.37$	0.50	$t_{(28)}=-1.23$, $p=0.229$	$t_{(22)}=-1.44$, $p=0.164$	$F_{(2,72)}=1.75$, $p=0.18$	
OD category error	$V=215$, $p=0.02$	0.08	$V=293$ $p<0.001$	$V=191$, $p<0.001$	$\chi^2_{(2)}=4.96$, $p=0.08$	
OD item error	$t_{(22)}=2.15$, $p=0.04$	0.13	$t_{(24)}=4.63$, $p<0.001$	$t_{(19)}==3.87$, $p=0.001$	$F_{(2,65)}=1.49$, $p=0.23$	
OD spatial error	$t_{(22)}=0.12$, $p=0.91$	0.86	$t_{(24)}=-3.48$, $p<0.001$	$t_{(19)}=-1.48$, $p=0.156$	$F_{(2,65)}=2.40$, $p=0.10$	

*$p\leq0.05$, **$p\leq0.01$, ***$p\leq0.001$ (FDR adjusted). P values underwent FDR correction for multiple comparisons. OI=Objects immediate, OD=Objects delayed

	**Processing Speed**	**Objects memory**	**Executive Functions I**	**Executive Functions II**	**Language**	**Words Memory**
Word definitions	-0.01	0.14	0.01	0.18	**0.73**	0.15
Blocks	0.16	-0.01	0.20	**0.54**	0.07	0.05
Card Pairs	0.16	0.56	**0.58**	0.00	0.13	-0.11
Digit Span	0.17	0.10	0.13	0.06	**0.57**	-0.10
Emotion Discrimination	0.08	0.16	-0.18	**0.48**	0.15	0.17
Four Towers	0.11	0.13	0.37	**0.69**	0.05	-0.07

2D Manipulations	**0.55**	-0.06	0.31	-0.01	0.14	0.21
Motor Control	**-0.40**	0.05	-0.11	-0.09	-0.05	-0.22
Objects Delayed	0.08	**0.77**	0.11	0.19	0.10	0.33
Objects Immediate	0.10	**0.84**	0.08	0.18	0.11	0.26
Paired Associate Learning	0.03	0.08	**0.68**	0.04	0.17	0.11
Picture Completion	-0.19	-0.07	**-0.60**	-0.18	0.07	-0.22
Spatial Span	0.22	0.21	**0.52**	0.03	0.16	-0.10
Simple RT	**-0.62**	-0.08	0.03	-0.37	-0.10	-0.04
Stroop	-0.08	-0.09	**0.69**	0.33	0.11	0.13
Target Detection	**0.69**	0.09	0.10	-0.03	0.05	0.08
Tower of London	0.03	0.12	0.20	**0.54**	0.33	-0.02
Trail Making	**-0.52**	-0.36	-0.13	0.11	-0.40	0.16
Verbal Analogies	0.12	-0.05	0.20	0.23	**0.65**	0.23
Words Immediate	0.09	0.27	0.14	0.04	0.15	**0.71**
Words Delayed	0.10	0.14	0.07	0.04	0.00	**0.81**
Choice RT	**-0.64**	-0.20	0.06	-0.30	0.01	0.15

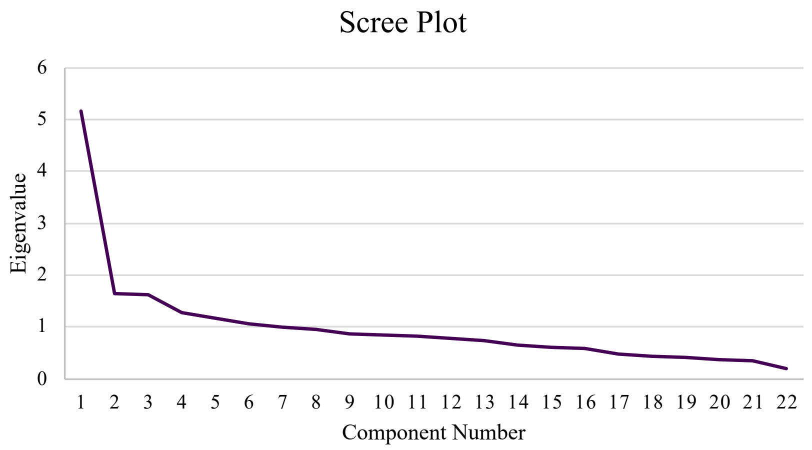

Supplementary figure 1. Results of the principal component analysis applied to the Cognitron tasks. Top. Rotated component matrix showing the loadings of each task on the extracted components. The

component to which each task was assigned for the computation of composite scores is indicated by the loading shown in bold. Bottom. Scree plot showing the eigenvalues extracted from the principal components analysis.

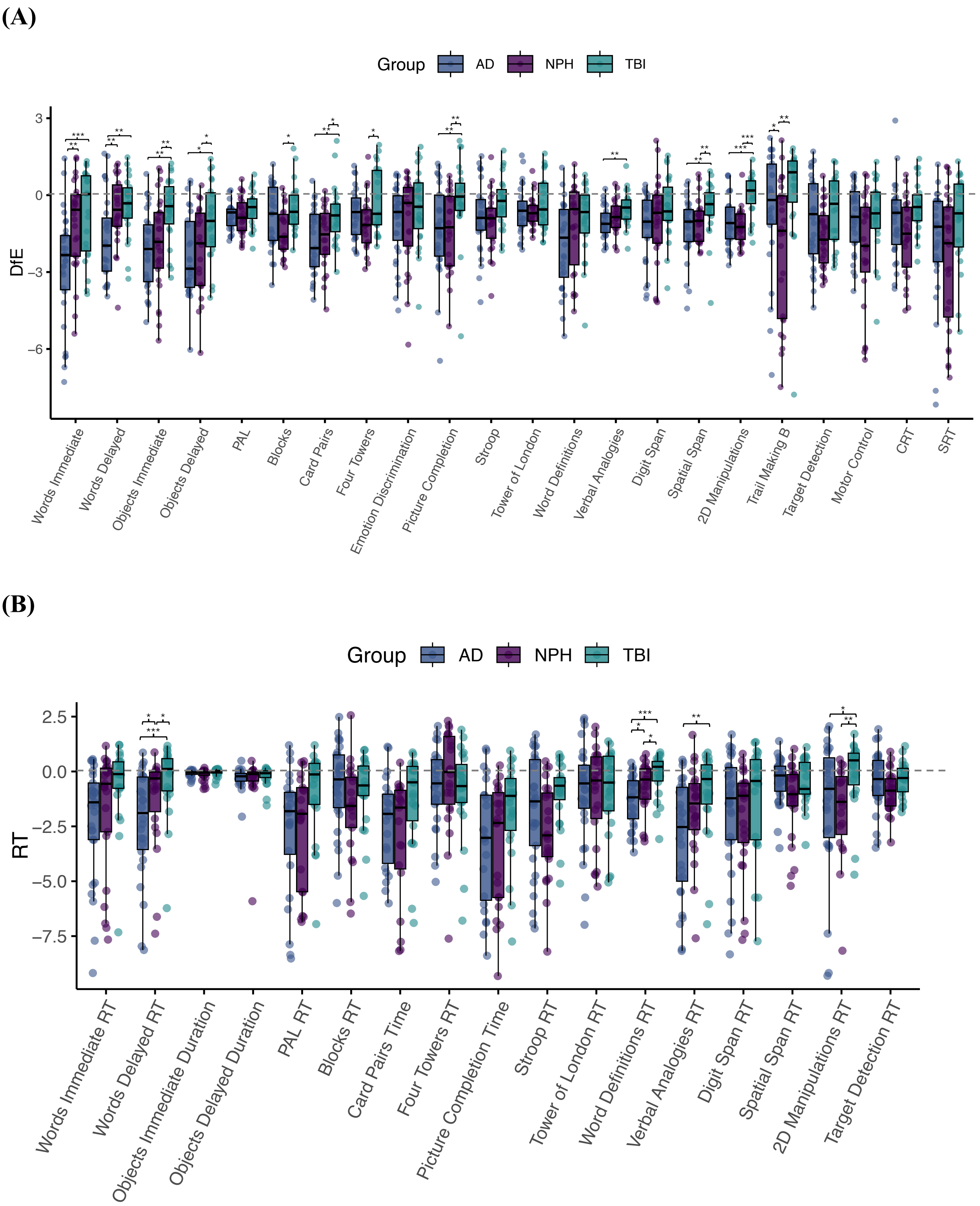

Supplementary Figure 2. Between-group comparisons of summary measures (A) and reaction time (B) scores in Deviation from Expected (DfE) format. The dotted line represents the expected performance for an individual with the same demographic characteristics as the patient but no clinical diagnosis. *p≤0.05, **p≤0.01, ***p≤0.001 (FDR adjusted).
